# An Update on the Morphology and Phylogeny of the Nanoplanktonic Dinoflagellate *Prorocentrum nux*

**DOI:** 10.1111/jeu.70019

**Published:** 2025-06-26

**Authors:** Solenn Mordret, Jenna MacKinnon, Susana A. Breglia, Claudio H. Slamovits, Caroline Chénard

**Affiliations:** ^1^ Aquatic and Crop Resource Development‐National Research Council Canada Halifax Nova Scotia Canada; ^2^ Department of Biochemistry and Molecular Biology Institute for Comparative Genomics – Dalhousie University Halifax Nova Scotia Canada

**Keywords:** Dinophyceae, Laurentian channel, plankton, Prorocentrales, protist, taxonomy

## Abstract

The Prorocentrales are a diverse group of dinophytes found in a wide range of marine environments. However, species delimitation in the genus *Prorocentrum* still remains a challenge, especially for nanoscale species where morphological and molecular information is often incomplete. *Prorocentrum nux* is an example where information is sparse and efforts are needed. In this study, we present a detailed description of the morphological features and molecular information for a Northwest Atlantic strain (AGSB‐0131). Using Scanning Electron Microscopy, additional morphological features from the original description were highlighted, including the periflagellar area configuration (nine vs. seven platelets) and pattern dissimilarities for the large and marginal pores. In addition to the revised description, the 18S rRNA gene, partial 28S rRNA gene, mitochondrial c*ox1* and *cob* genes were generated from strain AGSB‐0131, revealing some ambiguity in the existing data available for *P. nux* . An important genetic divergence between the only 18S rRNA sequence currently available for *P. nux* (strain RCC303) and AGSB‐0131 was highlighted, suggesting the importance of using the same strain to retrieve morphological and molecular information for nanodinoflagellates. Adding genetic markers for the correct identification of *P. nux* will enhance the ability to access its prevalence in the marine environment.

## 1 Introduction

Members of the genus *Prorocentrum* Ehrenberg exhibit very distinctive morphological features among dinoflagellates, making *Prorocentrum* cells stand out in plankton samples. Those unique morphological characteristics include the presence of two large thecal plates encapsulating the cell with a marked intercalary band suture, a periflagellar area with clustered platelets along the accessory pore, and the absence of both cingulum and sulcus, resulting in a desmokont flagellation (i.e., the two flagella inserted in an apical position through the flagellar pore). Within the genus *Prorocentrum*, taxonomic delineation between the different species has been an ongoing challenge due to morphological plasticity between members of the genus and the limited resolution of traditional taxonomic approaches. Morphological characterization of *Prorocentrum* species is historically based on the following features: (i) shape and size of the cell; (ii) thecal plate ornamentations; (iii) number and arrangement of platelets (typically 5–14 platelets); (iv) presence, size, and number of thecal pores; and (v) presence of thecal protrusions in the apex of the cell (Hoppenrath et al. 2013; Tillmann, Gottschling, et al. 2023; Tillmann, Wietkamp, et al. 2023).

While there are currently 86 described and accepted species names of *Prorocentrum* (Guiry and Guiry 2025 as of March 28, 2025), many members of that genus still remain poorly characterized or unknown. Many species described before 1980 still lack a detailed description of the number and arrangement of platelets, a valuable taxonomic trait for distinguishing among species. Reconciliation of detailed morphological features with molecular information is also lacking for half of the members of *Prorocentrum* (Guiry and Guiry 2025—as of March 28, 2025), which could hinder proper taxonomic identification. This is especially true for the nano‐prorocentroid species (< 20 μm), which remain difficult to characterize morphologically due to their smaller size (Tillmann, Gottschling, et al. 2023; Tillmann, Wietkamp, et al. 2023). Over the past three decades, 23 new species of *Prorocentrum* have been named and identified, with multiple studies specifically describing nanoscale species (Guiry and Guiry 2025). Those nano *Prorocentrum* species recently described include *P. pervagatum* (Tillmann, Wietkamp, et al. 2023), *P. spinulentum* (Tillmann, Gottschling, et al. 2023) and *P. thermophilum* (Gómez et al. 2023). In addition to the discovery of new species, the nomenclature of many common *Prorocentrum* species has been revised or clarified using molecular markers. Several nano *Prorocentrum* species, including *P. ovum* , *P. pusillum* , *P. nanum* , and *P. sphaeroideum*, were first described by Schiller (1918) at the beginning of the 20th century. However, upon revision and with phylogenetic analysis, many of these species were often indistinguishable when morphological or molecular data were absent or incomplete.

*Prorocentrum nux* serves as another example of a described nano *Prorocentrum* species for which published studies combining both morphological and molecular information are notably lacking. The first known culture of *P. nux* was a strain isolated in 1957 near Plymouth (United Kingdom) and was first deposited at the UTEX Culture Collection as *Exuviaella pusilla*, then renamed as *Prorocentrum nanum* (strain UTEX LB 1008—Puigserver and Zingone 2002). Two other *P. nux* strains were isolated from two different regions of Mediterranean Sea: the coast of Naples in Italy (strain Pronap1) and Ligurian Sea in France (strain RCC303). The original description of *P. nux* by Puigserver and Zingone (2002) characterized the species as having an irregularly oval cell without an apical spine. Its thecal plates were described as smooth, containing few pores of varying sizes, and seven periflagellar platelets surrounding the flagellar pore.

While *P. nux* has been described based on the morphological observations captured by light microscopy (LM), scanning electron microscopy (SEM) and/or transmission electron microscopy (TEM) for the strains UTEX LB 1008 and Pronap1 (Puigserver and Zingone 2002), no molecular information associated with *P. nux* exists for either strain (see Table 2; Tillmann, Gottschling, et al. 2023; Tillmann, Wietkamp, et al. 2023). The first genetic information for this species came from sequencing the 18S rRNA sequence (KT860951) from strain RCC303 (Tillmann, Gottschling, et al. 2023; Tillmann, Wietkamp, et al. 2023), although complete morphological documentation was not provided. Similarly, in February 2023, other sequences attributed to *P. nux*, including the ITS (OQ383703) and 28S rRNA (OQ383739) from an uncharacterised strain BS3‐F8, were deposited in GenBank (Dzhembekova and Tillmann, unpublished). Considerable uncertainty remains when identifying small prorocentroid species without reliable sequencing reference information associated with quality morphological analyses for material comparison. Given the small size of nano *Prorocentrum* species and the challenge to collect morphological information for them, several authors have considered those species to be conspecific over time, even if some evidence has indicated that they should be kept separate. Providing updated reference data on morphology and molecular information by sequencing universal genetic markers for well characterized *P. nux* isolates would aid in the accurate detection of the species in environmental surveys and thus give insight into its temporal and spatial distribution (Caron 2013).

This study aims to integrate and validate all available data on *P. nux* by combining the morphological description and molecular characterization of strain AGSB‐0131, a small *Prorocentrum* strain isolated from the Northwest Atlantic waters and identified as *P. nux* . The molecular characterization provides the sequencing of genetic markers, including the 18S rRNA gene, partial 28S rRNA gene (D1–D3), and mitochondrial cytochrome b (*cob*) and cytochrome c oxidase (*cox1*) genes. By combining morphological and molecular approaches for the same strain, we reconcile existing data on *P. nux* and provide accurate genetic markers that will contribute to broader knowledge of the prevalence of *P. nux* in marine environments.

## 2 Materials and Methods

### 2.1 Sampling, Cell Isolation and Cultivation

Strain AGSB‐0131 was isolated from a seawater sample collected in the Laurentian Channel (45.983369 N and 57.516912 W) in the Northwest Atlantic Ocean with a CTD‐Rosette at the chlorophyll maximum depth (41 m) on 4th of August 2021. *Prorocentrum nux* was isolated by dilution series and further single‐cell isolation using L1 media (NCMA/Bigelow Laboratory for Ocean Sciences, USA). Once monoclonal, the strain AGSB‐0131 was deposited in the Algal Genomic and Synthetic Biology (AGSB) culture collection maintained by the National Research Council of Canada in Halifax, Nova Scotia. The culture is maintained in a seawater L1 medium (NCMA/Bigelow Laboratory for Ocean Sciences, USA, 34 ppt) at 16°C on a 16:8 (light: dark) cycle under 30 μmol of light. Culture of strain AGSB‐0131 is available upon request to the corresponding author.

### 2.2 Growth Rate Experiment

For the growth experiment, AGSB‐0131 was cultured in triplicate by inoculating three flasks containing 500 mL of sterile L1 media with 1.5 mL of culture each. After inoculation, a sample of 5 mL was immediately taken from each flask and every 24 h for 16 days. Each sample was used for enumerating phytoplankton cells with flow cytometry.

For flow cytometry analysis, 980 μL of each culture sample (in duplicate) was fixed using 20 μL of 25% EM glutaraldehyde (Sigma‐Aldrich, USA) and incubated at 4°C for 15 min. Samples were then flash‐frozen in liquid nitrogen and stored at −80°C until analysis. Flow cytometry was performed with the BD Accuri CSampler Plus (Becton Dickinson, USA) where each sample was run for 5 min at a rate of 35 μL min^−1^ with a threshold set at 800 on red fluorescence (FL3‐H). Data was analyzed with the BD Accuri C6 Software, where phytoplankton cells were enumerated based on the cytograms of size scatter versus fluorescence signal.

### 2.3 Light Microscopy

Live‐cell imaging and observations were acquired using a light microscope (BX63 Light, Olympus, USA) and an inverted light microscope (Eclipse 80i, Nikon, USA), and both operated with epifluorescence and cameras (Olympus BX63Light: DP28 color and DP23M monochrome) (Nikon Eclipse 80i: Nikon 4k Low Light Camera). To observe the thecal plates, some cells previously fixed in 1% glutaraldehyde (25% EM glutaraldehyde ) were stained using calcofluor white (Sigma‐Aldrich, USA) following Fritz and Triemer (1985).

### 2.4 Scanning Electron Microscopy

In order to concentrate cells for SEM, 30 mL of exponential culture (~10 days post inoculation) was centrifuged at 3200×*g* for 5 min (Sorvall ST 16R ThermoFisher Scientific), supernatant was removed by pipetting and the pellet was resuspended with 10 mL of fresh L1 media. Cells were then fixed by adding 100 μL of Lugol Iodine solution (Sigma‐Aldrich, USA), then gently mixed by tube inversion and stored at 4°C overnight. The next day, cells were collected on a 3‐μm pore size polycarbonate filter (Millipore Sigma) positioned in a Swinnex holder (Millipore Sigma) and rinsed 3 times with Milli‐Q water. Cells on filters were then dehydrated with a series of ethanol washes (30%, 50%, 70%, 80%, 90%, 95%, and three times at 100%). The filters were dried with liquid CO_2_ in a Leica EM CPD300 critical point drier for 4 h, following manufacturer instructions. Finally, the filters were mounted on a stub, sputter coated with gold/palladium in a Leica EM ACE600 sputter coater, then visualized with a Zeiss Sigma 300 FESEM Gemini scanning electron microscope (Dalhousie University—Electron Microscopy Core Facility, Halifax, Canada). Detailed measurements of fine structures in AGSB‐0131 cells were then taken based on SEM images using ImageJ (Schneider et al. 2012). Images included in this article were arranged in Inkscape (v. 1.1.2—https://inkscape.org). All SEM images taken for strain AGSB‐0131 were deposited on the BioImage Archive (EMBL‐EBI) and can be viewed following this link https://www.ebi.ac.uk/biostudies/bioimages/studies/S‐BIAD1496 (doi: 10.6019/S‐BIAD1496).

### 2.5 DNA Extraction, Amplification and Sequencing

Genomic DNA was extracted from 10 mL of culture using the DNeasy Plant Pro Kit (Qiagen, USA) and eluted in 75 μL of EB buffer, following the manufacturer's instructions. The extracted DNA was stored at −20°C until further analysis.

The full 18S rRNA gene, partial 28S rRNA gene (D1–D3 domain), and mitochondrial cytochrome b (*cob*) and cytochrome c oxidase (*cox1*) genes were amplified and Sanger sequenced using universal eukaryotic primer sets (Table S1). All PCR amplifications were carried out using LongAmp Taq 2× Master Mix (New England Biolabs, USA) following manufacturer guidelines. After visualizing bands by electrophoresis in a 1.2% gel, Sanger PCR amplicons were cleaned by column purification (Nucleospin—PCR clean up and gel extraction, Macherey‐Nagel, USA) and eluted in 25 μL of nuclease‐free water. Sanger sequencing was conducted in both directions using amplification primers for 18S rRNA, 28S rRNA, *cob*, and *cox1* genes (Table 1) by GENEWIZ (NJ, USA). Three additional internal primers were also used to cover the total length of the 18S rRNA (Table S1).

**TABLE 1:** Measurement (μm) of different characteristics of strain AGSB‐0131. The measurement was calculated from images taken with SEM in this study.

	*n*	Average	Median	Min.	Max.	SD
Cell length	65	7.12	7.15	6.21	8.13	0.38
Cell width	75	6.06	6.06	5.41	6.75	0.28
Cell depth	65	5.58	5.61	4.56	6.87	0.56
Periflagellar area width	60	1.91	1.93	1.35	2.10	0.13
Periflagellar area depth	53	1.25	1.27	0.99	1.46	0.11
Platelets holes size	3	0.06	0.07	0.05	0.07	0.01
Flagellar pore length	29	0.70	0.70	0.44	0.80	0.06
Flagellar pore width	32	0.43	0.44	0.17	0.51	0.06
Accessory pore length	25	0.47	0.46	0.39	0.75	0.07
Accessory pore width	24	0.18	0.18	0.12	0.44	0.06
Large pores size	375	0.27	0.27	0.19	0.36	0.03
Small pores size	152	0.14	0.14	0.11	0.19	0.02
Marginal pores size	153	0.10	0.10	0.06	0.13	0.01
Space in between sutures (stitches) on intercalary band	122	1.09	1.08	0.55	1.97	0.26

Additionally, the sequencing of a large fragment of the rRNA was performed with Oxford Nanopore Technologies (ONT) MinION Flongle flow cell using published primer sets (Table S1) and following the same amplification, purification, and sequencing procedure described in Mordret et al. (2024). The large fragment includes a partial 18S rRNA gene (~1200 bp), the internal transcribed spacer region (ITS1, 5.8S rRNA gene and ITS2) and the partial 28S rRNA (i.e., approximately 900 bp).

### 2.6 Sequence Processing

Long‐reads obtained through nanopore sequencing were processed as previously described by Mordret et al. (2024). Contigs were built from Sanger sequences on Geneious Prime (v.2024.0.4) and identified using BLAST. All sequences were deposited on GenBank with accession numbers (PQ510849, PQ510850, PQ508261, PQ639436, PQ650243) (Table 2).

### 2.7 Molecular Phylogenies

For the phylogenetic analysis of 18S and 28S rRNA, all *Prorocentrum* sequences (txid2944[Organism:exp]) deposited on Genbank for both 18S (sequences more than 1000 bp) and 28S rRNA (sequences of ~900 bp) were downloaded (Genbank update—January 2024) and aligned using MAFFT (Katoh and Standley 2013) with AGSB‐0131 Sanger sequences. Fast trees were built for 18S rRNA and 28S rRNA genes individually to verify the taxonomic placement of AGSB‐0131 sequences. Species names as annotated on GenBank (i.e., the species names from the publication) were verified and corrected (if necessary) based on Algaebase (Guiry and Guiry 2025) to avoid ambiguity in the trees and to include the most up‐to‐date list of accepted *Prorocentrum* species (Table S5). A selective set of sequences was then chosen based on quality and schematic diversity criterion for 18S and 28S rRNA. Two matrices of 78 and 98 sequences were built including the selected set of sequences, AGSB‐0131 Sanger sequences, and *Azadinium* outgroup sequences (18S rRNA tree: HQ324898, JN680857 and LS974170; and 28S rRNA tree: LC756461, MW300580 and OQ379024). Matrices were aligned with MAFFT and trimmed to equal size up to 1738 and 747 bp for 18S rRNA and 28S rRNA, respectively. Phylogenetic analyses were performed on Geneious Prime (v. 2024.0.4) with phylogeny plugins PhyML and MrBayes. Additional phylogenetic analyses were carried out with IQ‐TREE (v 2.4.0, Minh et al. 2020), generating non‐parametric bootstrap with the ultrafast bootstrap method. Both 18S and 28S rRNA matrices were first tested with Modeltest (Darriba et al. 2012) and ModelFinder (with IQ‐TREE, Kalyaanamoorthy et al. 2017) to select the best evolution model. Robustness of tree topologies was assessed with 1000 replicates for Maximum Likelihood and IQ‐TREE phylogenies; and Bayesian inferences were calculated with 1,100,000 generations per run, four independent runs, 100,000 burn‐in length and 200 subsampling frequency.

The same process was used to select sequences and infer the phylogeny for the *cox1* mitochondrial gene. As a result, a matrix of 15 sequences was built including two *Scrippsiella* sequences ( *Scrippsiella sweeneyae* : EF036593 and *Scrippsiella* sp.: EF036592) which were used as outgroups to root the phylogeny. The matrix was aligned with MAFFT and trimmed to 976 bp.

A long‐read concatenated matrix including prorocentralean dinophyte sequences was built (21 sequences—3777 bp) where each of the rRNA genes (i.e., 18S, ITS1, 5.8S, ITS2 and 28S genes) were aligned using MAFFT. Phylogenetic analyses were performed with MrBayes under the GTR + gamma substitution model using the same parameters as described for the 18S and 28S rRNA phylogenies.

All trees produced in this study, including IQ‐TREE phylogeny, Maximum Likelihood bootstraps and Bayesian inferences, were rooted with the outgroup in Figtree implemented within Geneious Prime (v. 2024.0.2) and curated for visualization in Inkscape (v. 1.1.2—https://inkscape.org).

### 2.8 Geographical Distribution

The geographical distribution of AGSB‐0131 was assessed by searching the sequences of its V4 and V9 regions of the 18S rRNA gene against a database of metabarcodes (metaPR^2^, version 2.1.1) (Vaulot et al. 2022). The metaPR^2^ database contains a global oceanic data with more than 4150 samples originating from 41 published studies. The 18S rRNA V4 region (419 bp) and V9 region (129 bp) were extracted from the AGSB‐0131 sequences using universal V4 and V9 metabarcoding primers (Table S1) in Geneious Prime (v.2024.0.4) using the search and extract region tools. Once extracted, both regions were first assessed via Basic Local Alignment Search Tool (BLAST) on the NCBI reference database to check for similarity with other known V4 and V9 regions. Subsequently, both regions were used as queries in the web‐based application (https://shiny.metapr2.org). Only the ASVs with more than 99% similarity were kept and best result maps are shown in Tables S3 and S4 and Figures S6 and S7.

### 2.9 Terminology

All terminology in this article, including cell orientation, periflagellar area description, ornamentations, thecal plates, platelets, and pores, is based on other *Prorocentrum* cells reviewed by Hoppenrath et al. (2013) and Tillmann et al. (2019). The number of *Prorocentrum* species with molecular data associated with their names was reviewed by searching with the Genbank Taxonomy tool. Data available on *P. nux* —RCC303 can be accessed here https://roscoff‐culture‐collection.org/rcc‐strain‐details/303.

## 3 Results

### 3.1 Detailed Description

#### 3.1.1 Isolation and Growth Rate

Strain AGSB‐0131 was isolated from the Laurentian Channel (45.983369 N and 57.516912 W) at chlorophyll maximum depth (41 m) on August 4 2021. When cultivated in the laboratory at 16°C on a 16:8 (light: dark) cycle under 30 μmol of light, strain AGSB‐0131 exhibited a growth rate of 0.89 day^−1^ and reached stationary phase after 12 days, plateauing at an average 4.7 × 10^4^ cell mL^−1^ (*n* = 3).

#### 3.1.2 Light Microscopy

**FIGURE 1:**
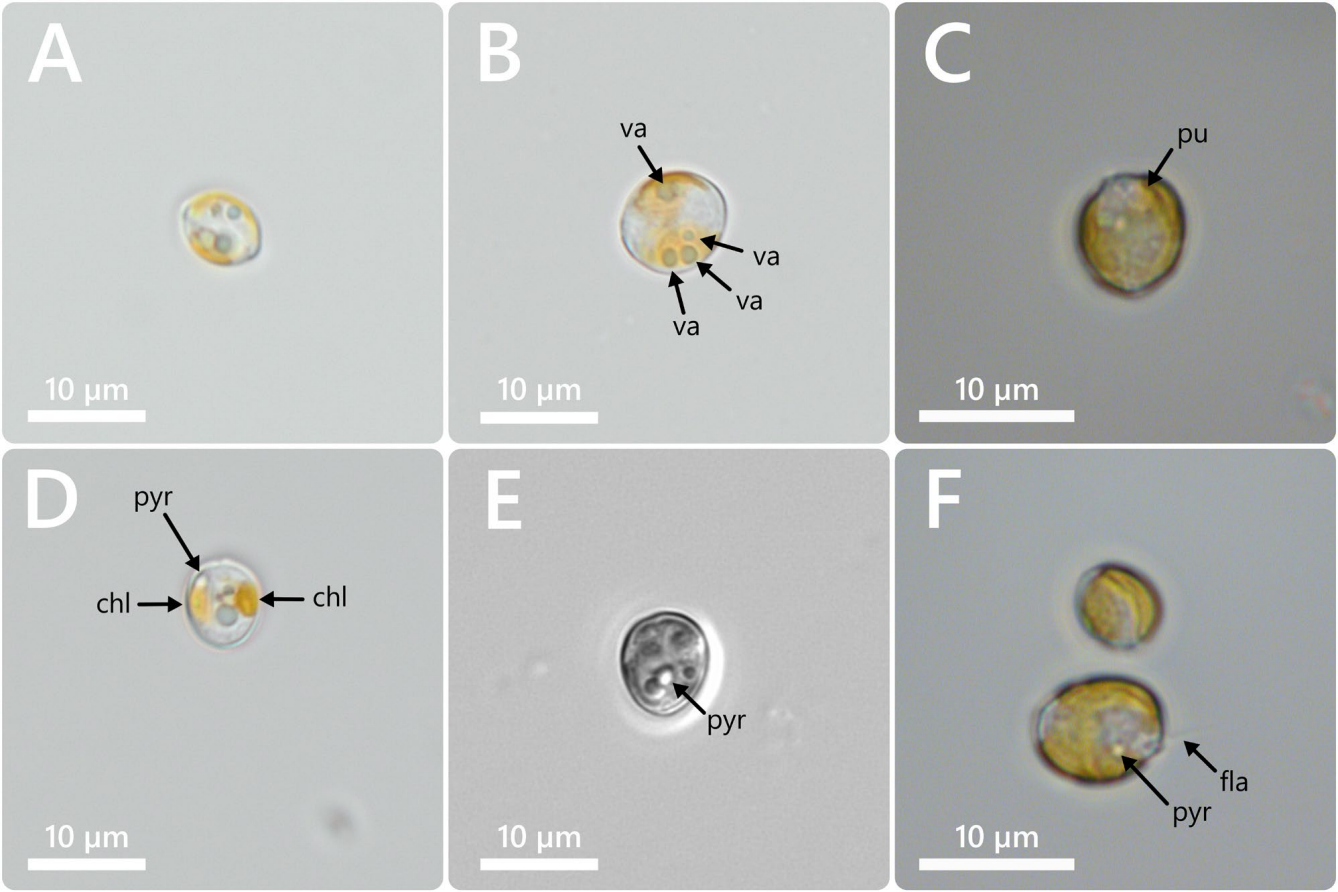
*Prorocentrum nux*, strain AGSB‐0131. LM of living cells (A–H). Cells are in the lateral view. chl: chloroplast, pyr: pyrenoid, va: vacuole, pu: pusule, fla: flagella.

Based on the LM analysis, cells were irregularly oval (Figure 1) and exhibited an oval shape in the lateral view (Figure 1A). Cell length ranged from 6.68 to 8.60 μm (mean 7.78 ± 0.40 μm, *n* = 25), cell width ranged from 6.36 to 6.99 μm (mean 6.78 ± 0.16 μm, *n* = 12) and cell depth ranged from 5.41 to 6.59 μm (mean 6.01 μm ±0.32 μm, *n* = 13).

**FIGURE 2:**
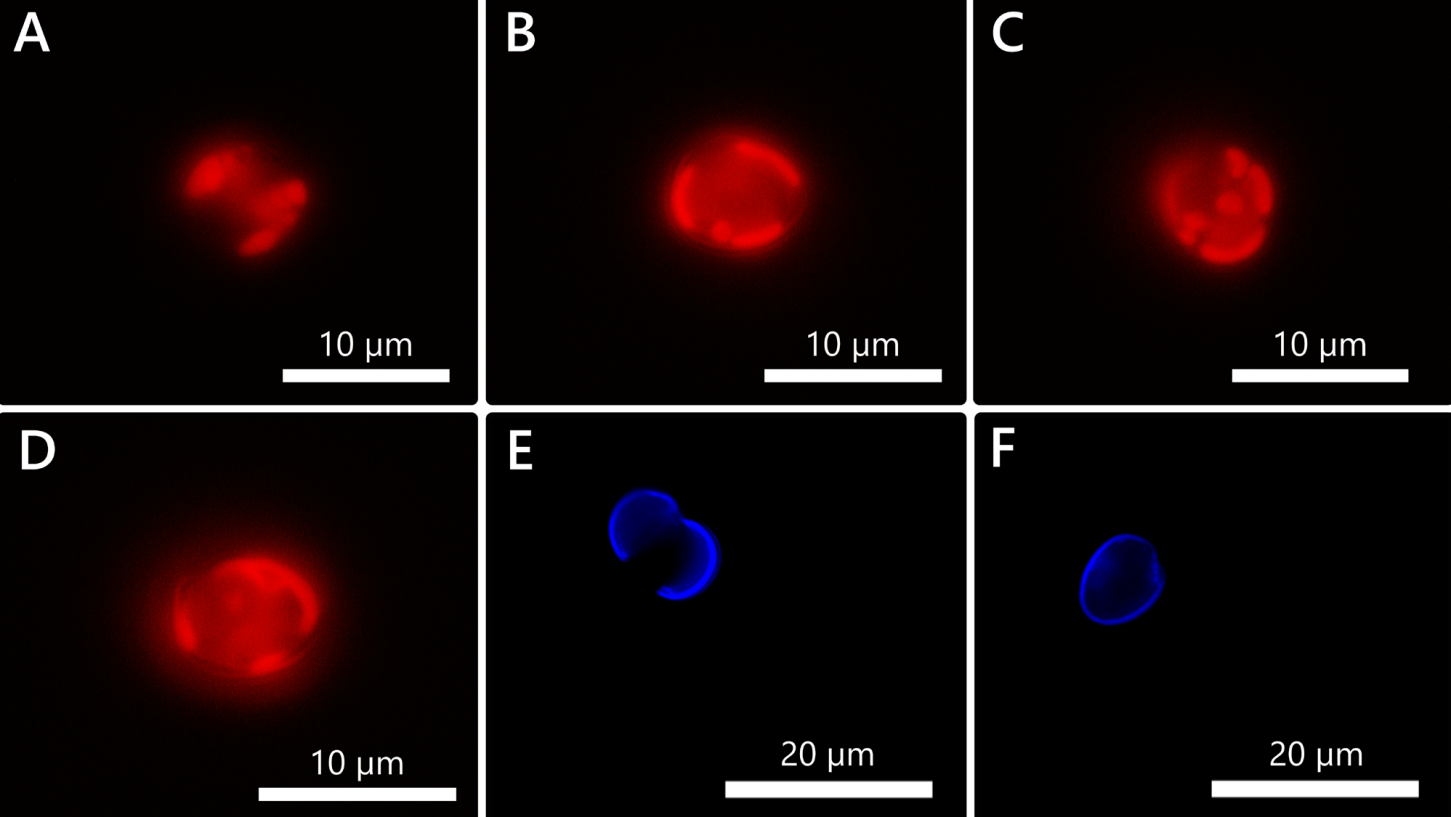
*Prorocentrum nux*, strain AGSB‐0131. Cell viewed with epifluorescence (A–D) or stained with calcofluor (E, F) under UV light excitation.

While the thecal plates were hard to detect under brightfield exposure (Figure 1), they were clearly visible in calcofluor‐stained cells under UV excitation (Figure 2). The thecae were very thin, smooth, and didn't display ornamentations. In contrast, the apical region of the cell with the periflagellar area was clearly visible due to the slight indentation in the valve shape in that zone (Figure 1). Additionally, two ochre‐yellow reticulate chloroplasts were arranged parietally and adjacent to the valves (Figure 1D), and several white and light green vesicles could sometimes be observed within the cells (Figure 1). A white pusule‐like structure could be found near that periflagellar area, and two matte white pyrenoid‐like vesicles were present next to the chloroplasts (Figure 1C–F). Light green vesicles observed within the cells were likely vacuoles (Figure 1B).

#### 3.1.3 Scanning Electron Microscopy

**FIGURE 3:**
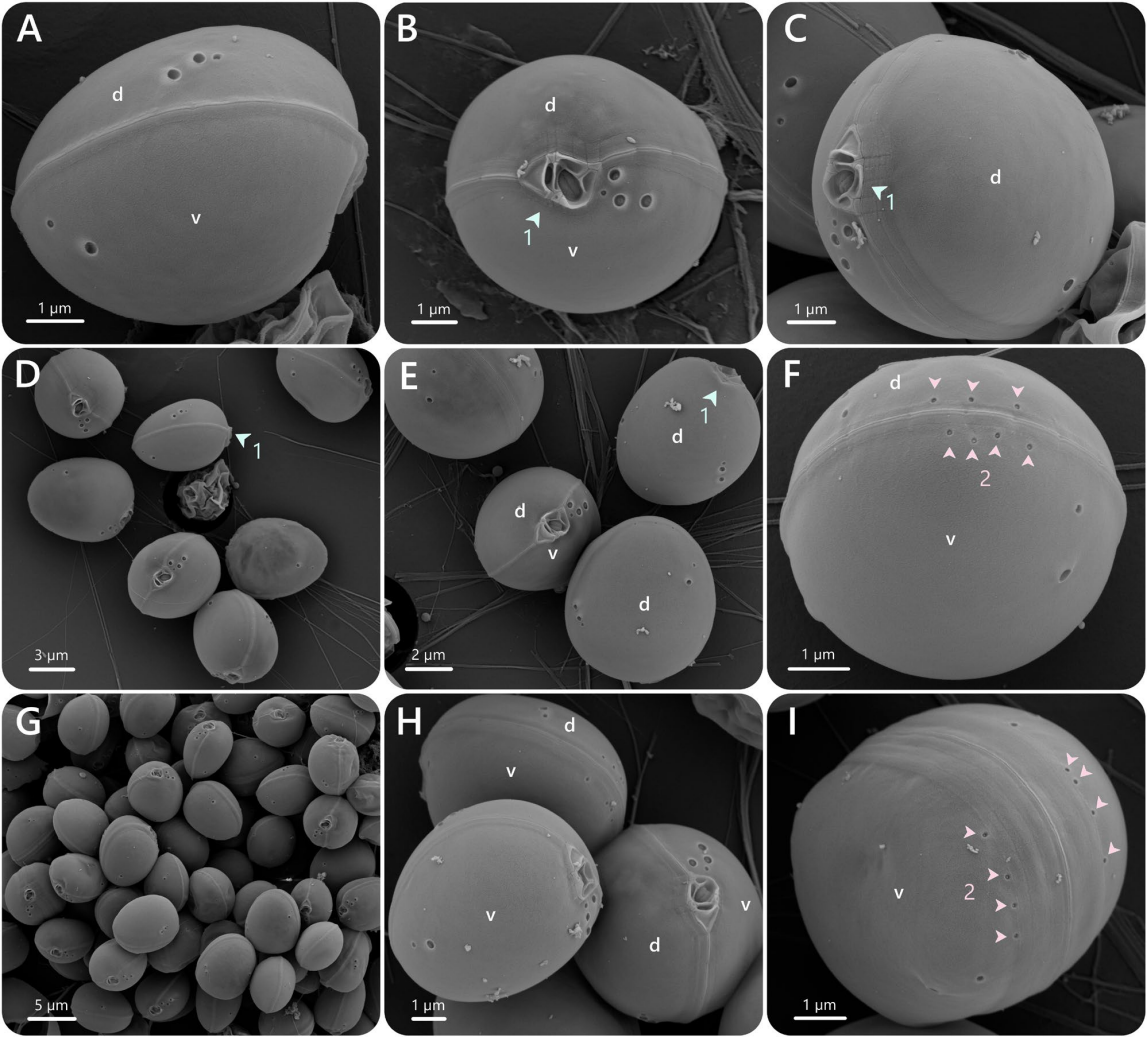
*Prorocentrum nux*, strain AGSB‐0131. SEM micrographs of different thecate cells. (d) dorsal valve, (v) ventral valve, (1) Periflagellar area, (2) marginal pores.

Based on the SEM analysis, the cells were round in the apical view and displayed an oval shape when viewed laterally (Figure 3A). Cell length ranged from 6.21 to 8.13 μm (mean: 7.12 ± 0.38 μm), cell width ranged from 5.41 to 6.75 μm (mean: 6.06 μm ± 0.28 μm) and cell depth ranged from 4.56 to 6.87 μm (mean: 5.58 μm ±0.56 μm) (Table 1, Table S2).

Like other prorocentroid species, cells were composed of two main cellulosic plates (i.e., valves). Ventral and dorsal valves were joined together in a sutured intercalary band, encapsulating the periflagellar area with its cluster of small platelets and flagellar pores on the apical part of the cell. The intercalary band presented a stitch‐like pattern and the distance between sutures ranged from 0.55 to 1.97 μm (mean: 1.09 ± 0.26 μm). The thecal plates were smooth and no apical spine was observed (Figure 3).

**FIGURE 4:**
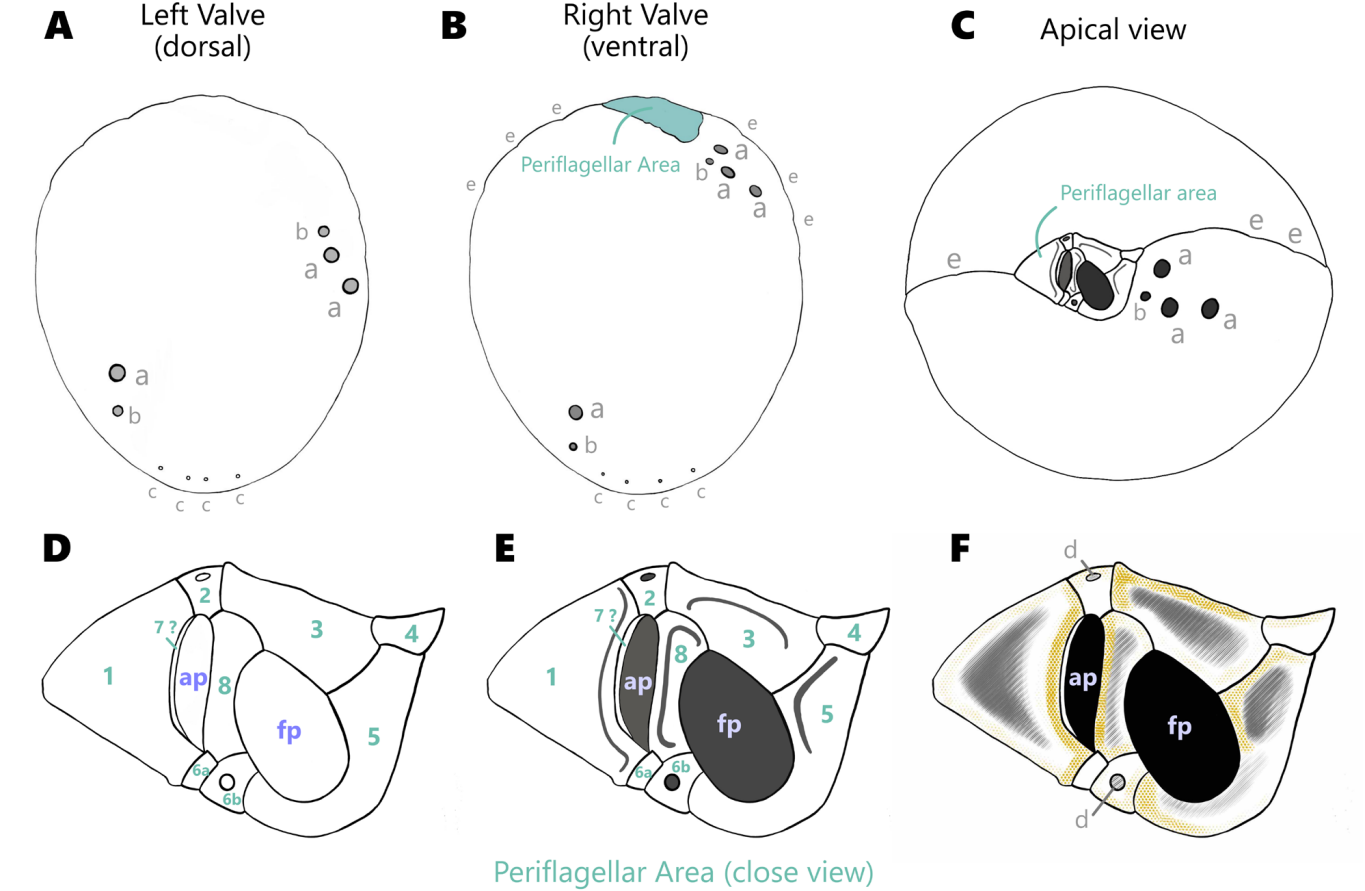
Schematic drawing of strain AGSB‐0131 with the representative pore patterns (a: large pore, b: small pore and c: marginal pore) for the right valve (A) and left valve (B). (C) Schematic drawing of the apical view of strain AGSB‐0131 with the representative pore patterns (a, b) and the suture of the intercalary band (e). (D) Schematic drawing of the periflagellar area showing the accessory pore (ap), the flagellar pore (fp) and Numbers (1–8) indicated the different platelets of the periflagellar area. (E) Schematic drawing of a close view of the periflagellar showing folding of the different platelets lists. (F) Schematic drawing of a close view of the periflagellar area topology displaying depression within the platelets in hatched gray including platelets “circular depressions” in (d), and higher lists patterns of platelets in dotted yellow.

**FIGURE 5:**
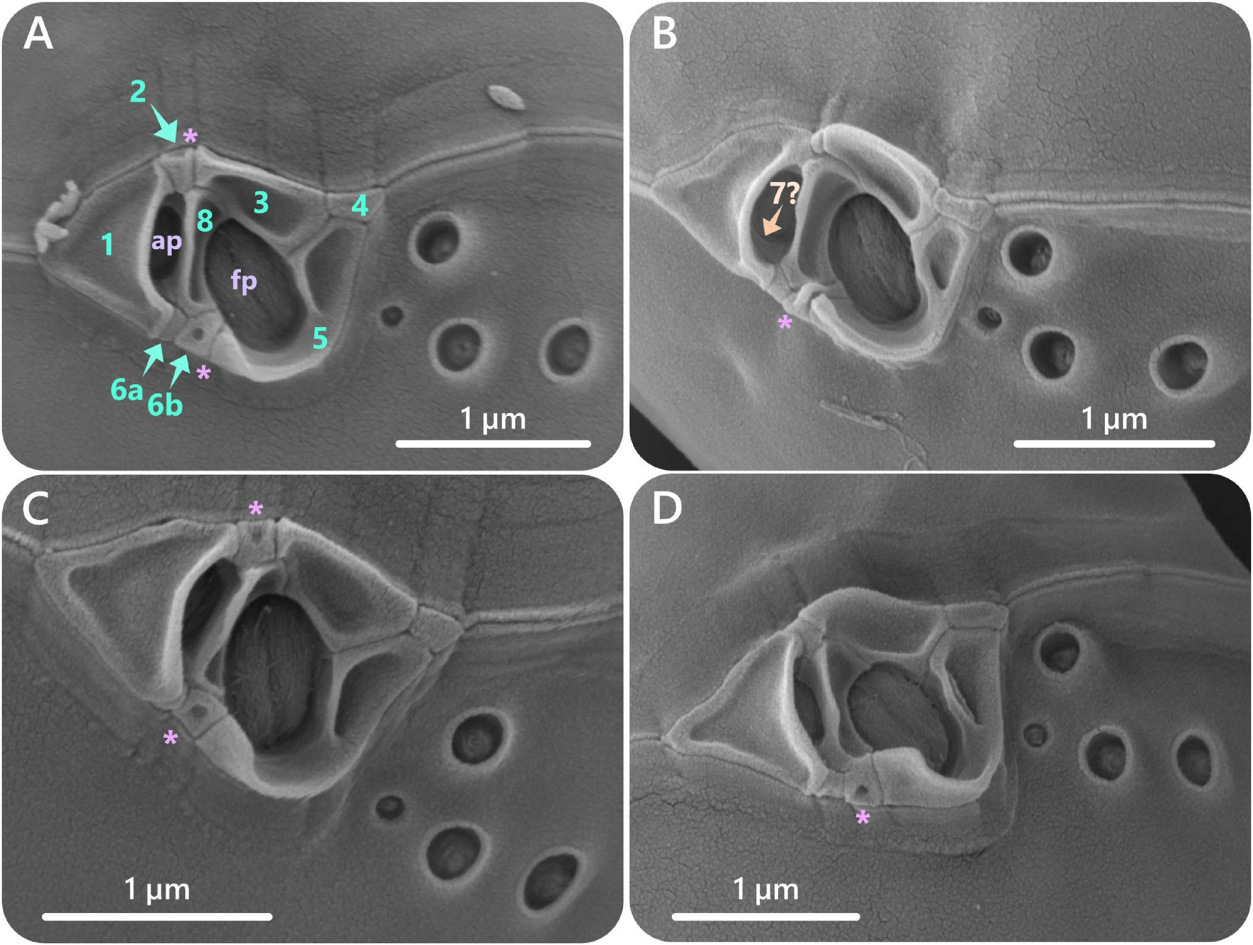
*Prorocentrum nux*, strain AGSB‐0131. SEM micrographs of the periflagellar area taken from different viewpoints (A–D). Platelets are annotated 1–8 in picture A. In micrograph B, potential platelet 7, visible inside the accessory pore (ap) and hidden by platelet 1, is annotated. Visible circular depressions are marked by an asterisk (*) in all micrographs (A–D).

The periflagellar area formed a wide V‐shaped structure and was nested on a slight indentation of the apical part of the ventral valve (Figures 3B–E, 4B and 5). The periflagellar area covered a region in width of 1.91 (±0.13 μm) and in depth of 1.25 (±0.11 μm) (Table 1, Table S2). The periflagellar area likely consisted of nine platelets (1, 2, 3, 4, 5, 6a, 6b, 7?, 8) surrounding the flagellar pore (fp) and the accessory pore (ap) (Figures 4C‐E and 5). The flagellar pore displayed a wide oval shape (length: 0.70 ± 0.06 μm and width: 0.43 ± 0.06 μm) and was bordered by platelets 3, 5, 6b and 8. Platelet 8 separated the flagellar and the accessory pores. The ap was smaller and narrower (length: 0.47 ± 0.07 μm and width: 0.18 ± 0.06 μm) and was surrounded by platelets 1, 2, 6a, 7 and 8. Platelet 7 was a very slim residual platelet, only visible around the left inside of the ap (Figure 5B, Figure S1A,B). Both flagellar and accessory pores were internally closed by two lip‐like structures (Figures 3B,C, and 5).

Several platelets showed extensions, depressions, and folds which were consistent throughout the cells (Figures 4D,E and 5). Platelets 1, 3, 5, and 8 showed a flat platelet list wrapped around the edges of each platelet and displayed a central depression (Figures 4F and 5). The right list of platelet 1 and left list of platelet 8 folded like small protrusions of two parallel structures protecting the ap. The lower part of platelet 5 displayed an external protrusion that formed a folded rim bending towards the flagellar pore. Platelet 4 was small in size and presented a plain surface, forming an inverted trapezoid shape on the apical right rim of the periflagellar area. Platelets 2, 6a, and 6b were of reduced size and were squeezed between the other platelets. Both platelets 2 and 6b regularly displayed a hole‐like circular depression in the center of the platelet.

Pores of different sizes and shapes were arranged at different places near the main plate margins. Larger pores were about 0.27 ± 0.03 μm in diameter while small pores were about 0.14 ± 0.02 μm in diameter. On the ventral valve, a cluster of one small and three large pores was located in the apical region (Figures 3B,C,E, 4B,C, 5, and Figure S1,S2). Those four apical pores were always located on the right side of the periflagellar area (ventral valve), with the small pore being the closest to platelet 5 and the other three big pores arranged in a triangular shape near platelets 4 and 5 (Figures 3B–E, 4C, and 5). A rare variation in the number of pores of the periflagellar area was observed on two occasions (*n* = ~250). The first variation showed two small pores (instead of one) near the periflagellar area (Figure S1C), while the second displayed two small pores and four big pores (instead of three) in the apical region (Figure S1D).

On the lower part of the ventral valve, one small and one large pore were also present in the left side (Figures 3 and 4B). On the dorsal valve, a row of two large pores and one small pore was visible near the right margin in the middle of the valve, while one large pore and one small pore were also present in the lower left part of the valve (Figures 3A and 4A). Those pores present in the middle or lower parts of the valves presented consistent patterns and were always arranged in the same order and number for all cells observed at SEM. A distinct row of three or four marginal pores (0.10 ± 0.01 μm) ran parallel to the intercalary band on both right and left valves (Figures 3F,I and 4A,B, Figure S2). Trichocysts could also be observed coming from large pores on a few pictures (Figure S1E,F).

#### 3.1.4 Molecular Characterization

The 18S rRNA gene (PQ510849:1765 bp), partial 28S rRNA gene (D1–D3 domain, PQ510850: 906 bp), mitochondrial c*ox1* (PQ639436: 1031 bp) and *cob* (PQ650243: 384 bp) genes were amplified and Sanger sequenced for Strain AGSB‐0131 (Table 2). A long gene fragment (PQ508261: 4287 bp) including the 18S (V4‐V9), ITS1‐5.8S‐ITS2 (thereafter ITS) and 28S (D1–D8) rRNA, was also sequenced using a MinION instrument (Oxford Nanopore MinION Technologies). Both Sanger and Nanopore sequences were compared and matched perfectly.

**TABLE 2:** Compilation of strain information, data collected, and reports in scientific literature available for *Prorocentrum nux*.

Strain name	Sample location	Coordinates	Sampling date	Sampling depth	Morphological data	Molecular data				
						18S rRNA	ITS rRNA	28S rRNA	cox1 (mit.)	cob (mit.)
AGSB‐0131^a^	Laurentian Channel, Canada	45.983369 N 57.516912 W	4 Aug. 2021	41 m (DCM)	LM, SEM	PQ510849 1765 bp		PQ510850 906 bp	PQ639436 1031 bp	PQ650243 384 bp
						PQ508261 4287 bp (MinION V4‐18S to D8‐28S rRNA)				
Pronap1^b^	Tyrrhenian Sea Mare Chiara station, Naples, Italy	40.816667 N 14.25000 E	23 June 1999	40 m	LM, SEM, TM					
UTEX LB 1008 = (Plymouth 184)^c^, ^d^	Plymouth water, UK	Unknown	1957	Unknown	LM, SEM, TM?					
RCC 303^e^	Gulf of Lion, France	43.000 N 7.000 E	10 Feb. 1998	50 m	LM, TM	KT860951 1807 bp				
BS3‐F8^f^	?	?	?	?	?		OQ383703 577 bp	OQ383739 691 bp		
E2:10K2^g^	San Matías Gulf, Argentina	42.001 S 64.932 W	March 2013	≈4 m	LM, SEM					

^a^ This study.

^b^ Puigserver and Zingone (2002).

^c^ Puigserver and Zingone (2002)*—* https://utex.org/products/utex‐lb‐1008.

^d^ DQ388459 18S rRNA sequence available for strain UTEX LB 1008 but now identified as *Exuviaella pusilla* (see Murray et al. 2009—Figure 2), EF036574 cox1 gene (partial cds) sequence was also available for strain UTEX LB 1008 but identified as *Exuviaella pusilla* and EU126136 cob gene (partial cds) sequence available for strain UTEX LB 1008 but identified as *P. nanum* (see Zhang et al. 2008)—Taxonomic names for those sequences were names reported on GenBank.

^e^ https://roscoff‐culture‐collection.org/rcc‐strain‐details/303.

^f^ https://www.ncbi.nlm.nih.gov/nuccore/KT860951.

^g^ Fabro and Almandoz (2021).

**FIGURE 6:**
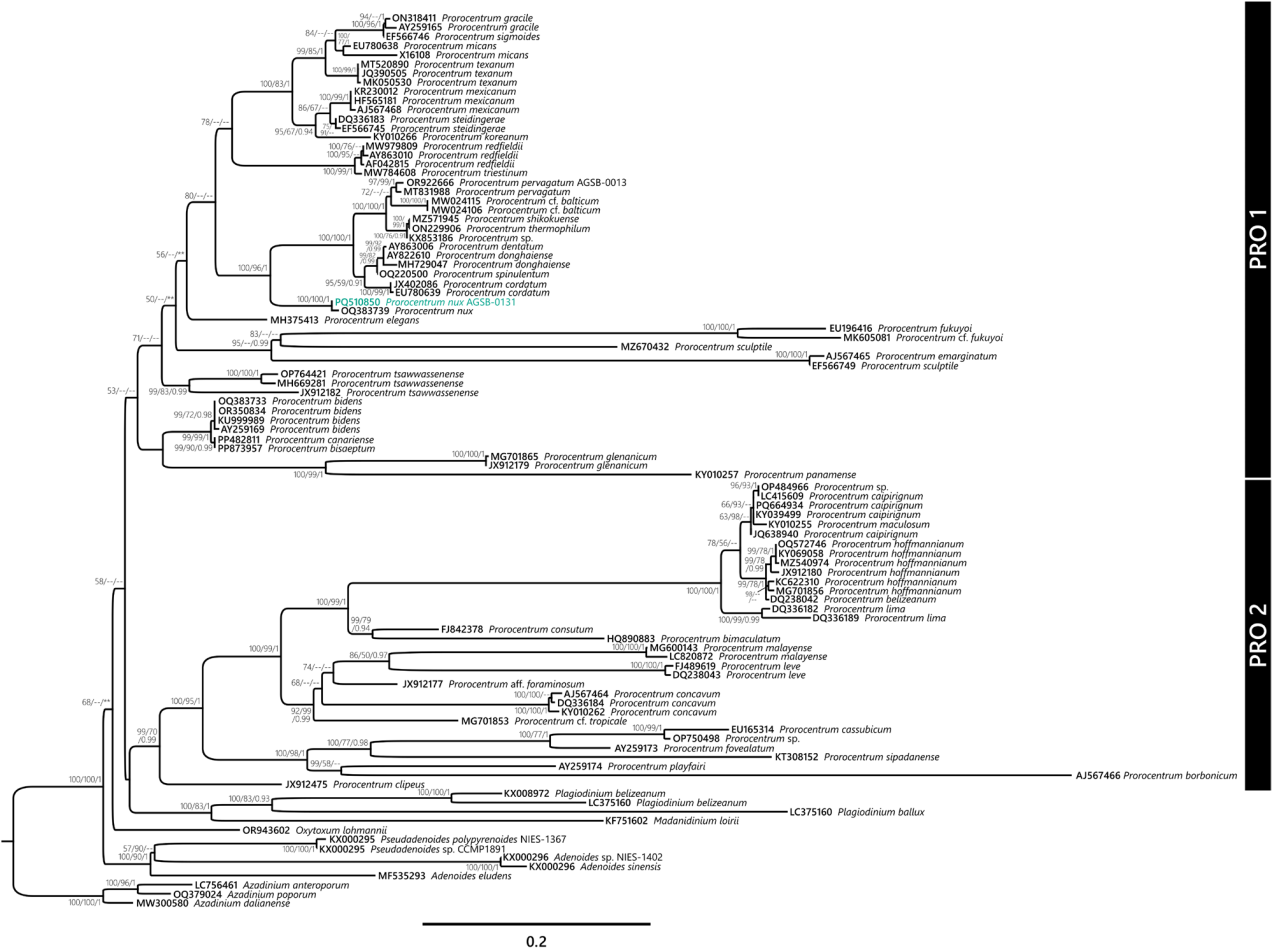
Molecular phylogeny prorocentralean dinophytes including the genus *Prorocentrum* based on the alignment of 28S rRNA. The strain AGSB‐0131 sequenced in this study is shown with the associated GenBank Accession Number in green. Corresponding PhyML bootstraps and Bayesian inferences were added to the tree at each node alongside iQtree bootstraps values. Bootstraps values and Bayesian inferences under 50 and 0.90, respectively, were annotated as ‐‐, and a different topology in the tree was annotated with **. For this phylogeny, sequences of *Azadinium anteroporum* (LC756461), *Azadinium poporum* (OQ379024) and *Azadinium dalianense* (MW300580) were used as outgroup.

Molecular phylogenetics based on the alignment of 18S and 28S rRNA genes resolved the members of the genus *Prorocentrum* into two main clades (e.g., PRO1 and PRO2) (Figure S3; Figure 6). The PRO1 included four previously described lineages: two lineages that include benthic species such as *P. tsawwassenense* and *P. fukuyoi*, one lineage for *P. micans* species complex and one lineage of nano *Prorocentrum*. Within this nano *Prorocentrum* clade, distinct clades were formed for *P. shikokuense*, *P. pervagatum*, *P. cordatum* (previously known as *P. minimum* ) and *P. nux* . Strain AGSB‐0131 formed a well‐supported clade with *P. nux* RCC303 (KT860951), in the 18S rRNA phylogeny (Posterior probabilities =1 and Bootstrap support = 100%—Figure S3) and a well‐supported clade with *P. nux* (OQ383739) in the 28S RNA phylogeny (IQtree Bootstrap 100 PhyML Bootstrap support = 100% and Posterior probabilities =1—Figure 6). However, the genetic divergence observed in the 18S rRNA marker between AGSB‐0131 and RCC303 showed 32 nucleotides mismatch (25 variable sites and seven gaps) between both sequences over a 1772 bp alignment. As a comparison, the number of mismatches observed between AGSB‐0131 sequence and other close species (such as *P. micans, P. cordatum, P. pervagatum* or *P. shikokuense*) was between 17 and 28 bp difference in the 18S rRNA alignment matrix. Specifically, the AGSB‐0131 V4 region had 5 mismatches with the RCC303 V4 region (KT860951) while the AGSB‐0131 V4 region only showed 3 mismatches with close related *Prorocentrum* species (*P. koreanum, P. mexicanum, P. micans, P. obtusidens* and *P. shikokuense*). The AGSB‐0131 V9 region showed 3 mismatches to the RCC303 V9 region (KT860951). For the ITS region and 28S rRNA gene, the difference between the reference sequences—OQ383703 (i.e., ITS‐5.8S‐ITS2 genes) and OQ383739 (i.e., 28S rRNA)—and the AGSB‐0131 nanopore‐derived sequence (PQ508261) displayed a difference of one gap in the ITS region and three bp differences in the 28S rRNA gene.

Phylogenetic analyses inferred from the mitochondrial *cox1* gene (Figure S4) showed strain AGSB‐0131 clustering with strains of *P. cordatum, P. pervagatum, P. mexicanum, P. micans*, and *P. texanum* with high bootstrap (i.e., BS = 99%) and probabilities support (i.e., PB = 1). Two strains of *P. cordatum* and *P. micans* clustered a sister clade. *P. lima* , *P. leve*, and *P. cassubicum* formed a more basal clade within the *Prorocentrum* genus. Very few references of *Prorocentrum* were available in public databases, and many species represented in 18S and 28S rRNA did not have corresponding *cox1* gene data (Figure S4). In the same way, the concatenated phylogeny built from available long‐length prorocentralean dinophyte sequences including the 18S, ITS1, 5.8S, ITS2, and 28S rRNA genes (Figure S5) showed that *P. nux* clustered as a sister clade to *P. pervagatum, P. shikokuense*, and *P. cordatum* (0.98 posterior probabilities). However, as per the *cox1* gene, few *Prorocentrum* sequences, including the 18S, ITS1, 5.8S, ITS2, and 28S rRNA genes from the same strains, were available in public databases.

#### 3.1.5 Geographical Distribution

Based on a BLASTn of the NCBI reference database (Sept 2024), both V4 and V9 regions were identified as unique, as no other known V4 and V9 regions were presently found to be 100% similar to the AGSB‐0131 sequences. The closest hit with the V4 region was *P. texanum* (MK995624, 99.28%), while the closest hit for the V9 region was *Amphidoma fulgens* (LC788755). The geographical distribution analysis of AGSB‐0131 using the V4 and V9 regions of the 18S rRNA gene against the metaPR^2^ database (Vaulot et al. 2022) assigned five and four ASVs (with at least 99% ID) for each region, respectively (Tables S3 and S4). Within the ASVs assigned to the AGSB‐0131V4 region, only one (ASV_b3327903cf) demonstrated a zero mismatch. This ASV was found in 34 samples with the highest abundance (~0.5% of eukaryotes) in a sample collected off the coast of South Africa (Figure S6). Within the ASVs assigned to the AGSB‐0131 V9 region, two ASVs (ASV_602b622101 and ASV_416a29cb7a) had 0 mismatch with the V9 region from AGSB‐0131 V9. Both ASVs were previously assigned to *Heterocapsa niei* on the metaPR^2^ database. While one ASV (ASV_602b622101) was found in 134 samples including samples collected off the coast of South Africa, Chile, and Madagascar, as well as in the Arabian Mediterranean and Red seas (Figure S7), one ASV (ASV_416a29cb7a) was only found in two samples from the Mediterranean Sea (Figure S7).

## 4 Discussion

### 4.1 Morphological Comparison

Light microscopy and SEM confirms the morphological taxonomic classification of AGSB‐0131 as *P. nux* , a species previously described by Puigserver and Zingone (2002). AGSB‐0131 displays the irregularly oval shape characteristic to *P. nux*. AGSB‐0131 cells were very similar in size (average = 7.78 μm) compared to Pronap1 and UTEX LB 1008 (i.e., 7.8 and 7.3 μm respectively) as measured by LM. However, the cell size was slightly smaller in the SEM measurement (7.12 μm), possibly due to a larger sample size or from the dehydration steps during the ethanol washes of the SEM preparation.

Features specific for the classification of *P. nux* could be re‐evaluated with the observation of AGSB‐0131 cells under the SEM. Structural details of the periflagellar area represent one of those specific characteristics, as higher‐resolution images revealed 9 platelets instead of 7 platelets identified in the original description (Puigserver and Zingone 2002). Firstly, platelet 6 (previously numbered as platelet 2 in Puigserver and Zingone 2002) could actually be divided into 2 distinct platelets (6a and 6b). The division of this platelet into 6a and 6b is a common feature for many *Prorocentrum* species (Hoppenrath et al. 2013), but was not yet observed in the periflagellar area of other described nanospecies. Secondly, a slim residual of platelet 7 was visible around the left inside of the ap on several AGSB‐0131 cells under the SEM. This was also observed by Tillmann and colleagues (2023) as an “overlooked” characteristic in the original description of *P. nux* . Despite not being reported by Puigserver and Zingone (2002), the presence of a residual platelet 7 in the inside left part of the accessory pore is a taxonomic characteristic in many closely related species, such as *P. pervagatum* (Tillmann, Wietkamp, et al. 2023), *P. spinulentum* (Tillmann, Gottschling, et al. 2023) and *P. cordatum* (Pei et al. 2022). However, the presence of nine periflagellar platelets (1, 2, 3, 4, 5, 6a, 6b, 7, 8) is uncommon among closely related nanospecies such as *P. pervagatum* (Tillmann, Wietkamp, et al. 2023), *P. spinulentum* (Tillmann, Gottschling, et al. 2023) and *P. cordatum* (Pei et al. 2022), in which eight platelets (1, 2, 3, 4, 5, 6, 7, 8) are generally enumerated. However, the periflagellar area and its platelet division for many nanoscale *Prorocentrum* species remain undescribed, and further work is still needed for many species.

Another structural feature that was previously overlooked for *P. nux* is the presence of a platelet hole‐like or circular depression on platelets 2 (previously numbered as platelet 5 in Puigserver and Zingone 2002) and 6b. This feature has not previously been reported, even though SEM pictures may show a similar structure on platelet 2 in the original description and on platelet 6b in Fabro and Almandoz (2021). In general, the periflagellar area of *P. nux* displays a similar profile to other species present in its sister clade with highly similar platelet composition, size, and shape. For example, a small platelet 4 was located in the right corner and a large platelet 5 surrounded the right side of the flagellar pore.

The pore patterns on the valves observed on the AGSB‐0131 cells were slightly different from the original description of *P. nux* (Puigserver and Zingone 2002). Unlike the original description of *P. nux* , three or four marginal pores instead of only three marginal pores ran parallel to the intercalary band on both valves. Apart from those marginal pores, the AGSB‐0131 pore pattern was similar to the original strain Pronap1. Cells observed by Fabro and Almandoz (2021) on a plankton sample also displayed the same pore pattern as AGSB‐0131. On the other hand, dissimilarity from strain UTEX LB 1008 could be observed, where one additional large pore was generally found on the left apical part of the dorsal valve and one additional pore was also located on the ventral valve. These variations in pore numbers and positioning are common among *Prorocentrum* species (Tillmann et al. 2019; Gómez et al. 2023; Tillmann, Gottschling, et al. 2023; Tillmann, Wietkamp, et al. 2023) and could represent plasticity within a species. Overall, a similar pore distribution near the valve edges and on the ventral right side of the periflagellar area is observed for most nanoscale *Prorocentrum* (Gómez et al. 2023; Tillmann, Gottschling, et al. 2023; Tillmann, Wietkamp, et al. 2023). Lastly, despite not being a specific taxonomic characteristic for *Prorocentrum*, it is interesting to highlight the unique stitch pattern on the intercalary band. This pattern was not included in the original description and is not commonly reported in other *Prorocentrum* species.

### 4.2 Genetic Comparison

As shown in the 18S and 28S rRNA phylogenies (Figure S3 and Figure 6), *P. nux* clustered as a sister clade of the nano *Prorocentrum* group with all the other known nanodinoflagellates (< 20 μm) including *P. dentatum, P. obtusidens, P. shikokuense, P. pervagatum, P. spinulentum*, and *P. cordatum*. Additionally, the concatenated phylogeny built from long‐read sequences confirmed the positioning among prorocentrealean dinophytes. Those findings were coherent with morphological observations previously reported (Tillmann, Gottschling, et al. 2023; Tillmann, Wietkamp, et al. 2023; Gottschling et al. 2024). However, unlike the historical morphological data, an important genetic divergence was observed between the first molecular information of *P. nux* derived from 18S rRNA sequence (KT860951, strain RCC303) and AGSB‐0131. A mismatch of 32 nucleotides (25 variable sites and seven gaps) between both sequences was observed over a 1772 bp alignment, suggesting species divergence as the mismatch was higher than that between AGSB‐0131 and other closely related species. The two variable regions (V4 and V9 regions) of the 18S rRNA gene mostly used to assign taxonomy in eukaryotes were particularly divergent for both *P. nux* strains, with more mismatches occurring between each other than closely related *Prorocentrum* species (*P. koreanum, P. mexicanum, P. micans, P. obtusidens* and *P. shikokuense*). The extracted V9 region sequence from strain RCC303 (KT860951) was not closely related to any dinoflagellates on a BLAST analysis (data not shown). Consequently, the discrepancy between both sequences might be a result of a sequencing error of the 18S rRNA from the culture RCC303 or a misclassification of the sequenced material as RCC303. In the same way, two sequences of 18S rRNA (DQ388459) and *cox*1 (EF036574) that were attributed to UTEX LB 1008 (now corrected and classified as *Exuviaella pusilla* on GenBank—Zhang et al. 2008) did not match sequences obtained for the AGSB‐0131 strain and cluster outside the *Prorocentrum* clade genus (Murray et al. 2009). On the other hand, a sequence of *cob* gene (EU126136) sequenced from UTEX LB 1008 (attributed to *P. nanum—*Zhang et al. 2008) matched perfectly with AGSB‐0131 *cob* gene sequence, adding to the confusion about the genetic information currently available for *P. nux* (see Table 2). The ambiguity in the genetic information of UTEX LB 1008 between the original description by Puigserver and Zingone (2002) and Zhang et al. (2008) suggested that the sequencing information: (i) might not have been from the same strain, (ii) were from two different species which suggested that UTEX LB 1008 and Pronap1 are two different species, or (iii) relied on data that did not meet the quality standard required for publishing reference gene information on public databases.

In contrast, recent sequencing of the ITS region (OQ383703) and 28S rRNA (OQ383739) from another strain, BS3‐F8—also assigned to *P. nux* (Dzhembekova and Tillmann, unpublished)—shows high similarity when compared to the corresponding sequences from AGSB‐0131, with only one gap in the ITS region and three mismatches in the 28S rRNA gene (over 578 and 666 bp comparison). As demonstrated in the 28S rRNA phylogeny (Figure 6), AGSB‐0131 and BS3‐F8 (OQ383739) form a strongly supported clade (BS/PP = 100/1), confirming that these two strains could be considered the same species.

### 4.3 Geographical Distribution and Putative Ecology of *P. nux*

Prior to this work, the known distribution of *P. nux* was only reported based on morphological identification from regions that included the coast of Plymouth (UK), Mediterranean Sea (Tyrrhenian Sea) and Argentina (Table 2—Fabro and Almandoz 2021; Tillmann, Gottschling, et al. 2023; Tillmann, Wietkamp, et al. 2023). Recent studies tentatively identified *P. nux* among the phytoplankton communities in plankton samples, despite not using molecular data to provide an accurate species identification (Cerino et al. 2012; Fabro and Almandoz 2021; Veselá‐Strejcová et al. 2023). Strain AGSB‐0131 represents the first report of *P. nux* from Northwest Atlantic waters. These reports suggest that *P. nux* could be a cosmopolitan species that thrives at various water temperatures. Like other known *P. nux* strains, AGSB‐0131 was isolated from the deeper part of the euphotic zone; strain RCC303 was isolated at 50 m depth and Pronap1 was isolated at 40 m depth. The slow growth rate (0.89 day^−1^) of strain AGSB‐0131 in the laboratory supports the assumption that *P. nux* is probably present in low abundance in the environment and therefore rarely reported in the field. However, field observations in the Argentina Sea have shown that *P. nux* is locally significant in the phytoplankton community and can reach concentrations up to 82,000 cells L^−1^ (Fabro and Almandoz 2021).

The geographical distribution of AGSB‐0131, based on both V4 and V9 regions of the 18S rRNA markers queried against the metaPR^2^ database, revealed the prevalence of *P. nux* like sequences in many other coastal areas, such as the South Africa coast and Chile, where *P. nux* may represent almost 0.5% of the protist communities sampled. The ambiguity in the 18S rRNA might have hindrance the detection of *P. nux* in environmental studies. For example, the V9 region from strain RCC303 (KT860951) didn't match any ASV (> 95% ID) against the metaPR^2^ database, which includes more than 4150 samples and 90,000 ASVs (Vaulot et al. 2022). The lack of an accurate reference sequence might also have led to errors in the previous taxonomic assignation of ASVs (i.e., assignation of V4 sequence to *Heterocapsa niei* and V9 to *P. shikokuense*) in the metaPR^2^ database. Both V4 and V9 regions for AGSB‐0131 are unique (based on a NCBI blast, September 2024) and differ from other known nanoscale *Prorocentrum* species, which support the ASVs identification. However, many known dinoflagellates species do not have unique V4 or V9 regions (Mordret et al. 2018; Le Bescot et al. 2016). For instance, *P. obtusidens*, *P. dentatum* and *P. shikokuense*, as well as *P. cordatum, P. rhathymum, P. micans* and *P. mexicanum* respectively share the same V4 region sequence. As several nanoscale *Prorocentrum* species are still to be isolated and characterized, it is also possible that those ASVs might represent other undiscovered *Prorocentrum* species closely related to *P. nux* . For example, Veselá‐Strejcová et al. (2023) identified with SEM an unknown smooth thecate *Prorocentrum* species from the Marquesas Islands which resemble *P. nux*, but with a larger cell size and a higher number of pores on both valves. Adding the accurate sequence information of *P. nux* to the reference dataset will facilitate the identification of *P. nux* in future environmental surveys and elucidate its importance in the marine environment.

By integrating both morphological and molecular identification, this work provides a comprehensive characterization of *Prorocentrum nux*, helping to address gaps in the historical data and clarify decades of puzzling molecular sequence information produced by various authors. Given the small size of the nano‐prorocentroid species, it is still challenging to provide a quick and accurate taxonomic classification without the use of genetic markers. Combining both morphology and molecular identification of poorly characterized nanospecies such as the overlooked *P. nux* species will facilitate future detection of that species in environmental studies and may highlight its prevalence in specific environmental conditions or demonstrate its cosmopolitan nature, as suggested by its various observations.

## Supporting information


**Data S1.** Primers used in this study. Primers used for nanopore sequencing included the MinION adapters (5′‐TTT CTG TTG GTG CTG ATA TTG C‐forward primer‐3′, 5´‐ACT TGC CTG TCG CTC TAT CTT C‐reverse primer‐3′).
**Table S2**. General characteristic of AGSB‐0131 based on the description of species by Puigserver and Zingone (2002).
**Table S3**. Assignment of the V4 region (add length) for *P. nux* from using the metaPR2 database. The table shows the ASV retrieved with at least 99% ID.
**Table S4**. Assignment of the V9 region (add length) for *P. nux* from using the metaPR2 database. The table shows the ASV retrieved with at least 99% ID.
**Figure S1**. SEM images of *P. nux* (AGSB‐0131) showing A & B a view of small platelet 7 (see arrow annotated as no. 1), partially covered/hidden by platelet 1; C & D the only two examples of cells observed in SEM with unusual variations in apical pore patterns and numbers (see arrow no. 2 showing an additional small pores and arrow no. 3 pointing at additional large pores) in the right apical portion of the ventral valve. SEM pictures E & F show trichocysts presence (shown by arrow no. 4).
**Figure S2**. SEM images of *P. nux* (AGSB‐0131) showing A an inside view of an empty ventral (v) valve, B an inside view of an empty dorsal (d) valve and C external view of a detached ventral valve. Pores were indicated with an asterisk and marginal pores with a sharp sign (#). Periflagellate area was annotated as “pa”. Scale bar is 1 μm.
**Figure S3**. Molecular phylogeny of prorocentralean dinophytes including the genus *Prorocentrum* based on the alignment of 18S rRNA. The strain AGSB‐0131 sequenced in this study is shown with the associated GenBank Accession Number in green. Corresponding iQtree bootstraps values were added to the tree at each node alongside Bayesian inferences. Bayesian support values and iQtree bootstraps values under 0.90 and 50, respectively, were annotated as – and a different topology in the tree was annotated with **. For this phylogeny, sequences of *Azadinium poporum* (HQ324898), *Azadinium spinosum* (JN680857) and *Azadinium trinitatum* (LS974170) were used as outgroup.
**Figure S4**. Molecular phylogeny of the genus *Prorocentrum* based on the alignment of (COI) mitochondrial gene. The strain AGSB‐0131 sequenced in this study is shown with the associated GenBank Accession Number in green. Corresponding RAxML bootstraps were added to the tree at each node alongside Bayesian inferences. Bayesian support values and ML bootstraps under 0.90 and 50, respectively, are annotated as –. For this phylogeny, sequences of *Scrippsiella sweeneyae* (EF036593) and *Scrippsiella* sp. (EF036592) were used as outgroup.
**Figure S5**. Bayesian Concatenated phylogeny of the genus *Prorocentrum* based on the alignment of the 18S, ITS1, 5.8S, ITS2 and 28S rRNA. The strain AGSB‐0131 sequenced in this study is shown with the associated GenBank Accession Number in green. Bayesian support values under 0.90 were not shown in the phylogeny. For this phylogeny, sequences of close related genera *Oxytoxum* and *Amphidoma* were also added. *Heterocapsa pseudotriquetra* (OP968025), *Heterocapsa steinii* (MF423346) and *Heterocapsa horiguchii* (OP970968) were used as outgroup.
**Figure S6**. Distribution of the amplicon sequence variant ASV_b3327903cf (V4 region) from the metaPR^2^ database (Vaulot et al., 2022). ASV_b3327903cf shown 100% similar with the V4 region from *P. nux* .
**Figure S7**. Distribution of the amplicon sequence variants (a) ASV_602b622101 and (b) ASV_416a29cb7a from the metaPR^2^ database (Vaulot et al., 2022). Both ASVs have 0 mismatch with the V9 region from *P. nux* .

**Table S5**. List of taxonomically accepted *Prorocentrum* species (Guiry and Guiry, 2025) with information on molecular data availability for each species.

## Data Availability

The data that support the findings of this study are openly available in GenBank at https://www.ncbi.nlm.nih.gov/genbank/, reference number (PQ510849, PQ510850, PQ508261, PQ639436, PQ650243).
