# Supplementary material for: An Update on the Morphology and Phylogeny of the Nanoplanktonic Dinoflagellate Prorocentrum nux
Source: J Eukaryot Microbiol. 2025 Jun 26;72(4):e70019. doi: 10.1111/jeu.70019 (PMC12198932; doi:10.1111/jeu.70019)
Supplement: Supplementary file 1 — Data S1. Primers used in this study. Primers used for nanopore sequencing included the MinION adapters (5′‐TTT CTG TTG GTG CTG ATA TTG C‐forward primer‐3′, 5´‐ACT TGC CTG TCG CTC TAT CTT C‐reverse primer‐3′). Table S2. General characteristic of AGSB‐0131 based on the description of species by Puigserver and Zingone (2002). Table S3. Assignment of the V4 region (add length) for P. nux from using the metaPR2 database. The table shows the ASV retrieved with at least 99% ID. Table S4. Assignment of the V9 region (add length) for P. nux from using the metaPR2 database. The table shows the ASV retrieved with at least 99% ID. Figure S1. SEM images of P. nux (AGSB‐0131) showing A & B a view of small platelet 7 (see arrow annotated as no. 1), partially covered/hidden by platelet 1; C & D the only two examples of cells observed in SEM with unusual variations in apical pore patterns and numbers (see arrow no. 2 showing an additional small pores and arrow no. 3 pointing at additional large pores) in the right apical portion of the ventral valve. SEM pictures E & F show trichocysts presence (shown by arrow no. 4). Figure S2. SEM images of P. nux (AGSB‐0131) showing A an inside view of an empty ventral (v) valve, B an inside view of an empty dorsal (d) valve and C external view of a detached ventral valve. Pores were indicated with an asterisk and marginal pores with a sharp sign (#). Periflagellate area was annotated as “pa”. Scale bar is 1 μm. Figure S3. Molecular phylogeny of prorocentralean dinophytes including the genus Prorocentrum based on the alignment of 18S rRNA. The strain AGSB‐0131 sequenced in this study is shown with the associated GenBank Accession Number in green. Corresponding iQtree bootstraps values were added to the tree at each node alongside Bayesian inferences. Bayesian support values and iQtree bootstraps values under 0.90 and 50, respectively, were annotated as – and a different topology in the tree was annotated with **. For this phylogeny, sequences of [file JEU-72-e70019-s001.docx]

# **An update on the morphology and phylogeny of the nanoplanktonic dinoflagellate *Prorocentrum nux***

**Supplementary Information**

**Table S1** - Primers used in this study. Primers used for nanopore sequencing included the MinION adapters (5´-TTT CTG TTG GTG CTG ATA TTG C-forward primer-3´, 5´-ACT TGC CTG TCG CTC TAT CTT C-reverse primer-3´).

| **Primer Name** | **FOR/REV** | **Sequence (5’ -> 3’)** | **Marker** | **Type** | **References** |
| --- | --- | --- | --- | --- | --- |
| **SSU-F_Adapt** | F | TTT CTG TTG GTG CTG ATA TTG C TC YAA GGA AGG CAG CAG GCG C | 18S | Nanopore | Hamsher et al., 2011 |
| **D3Ca-R_Adapt** | R | ACT TGC CTG TCG CTC TAT CTT C GA CGA SCG ATT TGC ACG TCA G | 28S | Nanopore / Sanger | modified from Scholin et al., 1994a |
| **SA** | F | CAA CCT GGT TGA TCC TGC CAG T | 18S | Sanger | Medlin et al., 1988 |
| **V4f** | F | CCA GCA SCY GCG GTA ATT CC | 18S-V4 | Sanger | Modified from Stoeck et al., 2010 |
| **V4r** | R | ACT TTC GTT CTT GAT YRA | 18S-V4 |  | Modified from Stoeck et al., 2010 |
| **1055R** | R | CTA AGA ACG GCC ATG CAC CAC CAC C | 18S | Sanger | Gaonkar et al., 2020 |
| **1510R (V9)** | R | CCT TCY GCA GGT TCA CCT AC | 18S-V9 | Sanger | Amaral-Zettler et al., 2009 |
| **D1R-C_m** | F | ACC CGC YGA AYT TAA GCA | 28S-D1 | Sanger | modified from Scholin et al., 1994b |

**Amaral-Zettler, L. A.,** McCliment, E. A., Ducklow, H. W., & Huse, S. M. (2009). A method for studying protistan diversity using massively parallel sequencing of V9 hypervariable regions of small-subunit ribosomal RNA genes. PloS one, 4(7), e6372.

**Gaonkar, C. C**., Piredda, R., Sarno, D., Zingone, A., Montresor, M., & Kooistra, W. H. (2020). Species detection and delineation in the marine planktonic diatoms *Chaetoceros* and *Bacteriastrum* through metabarcoding: making biological sense of haplotype diversity. *Environmental Microbiology*, *22*(5), 1917-1929.

**Hamsher, S. E.,** Evans, K. M., Mann, D. G., Poulíčková, A., & Saunders, G. W. (2011). Barcoding diatoms: exploring alternatives to COI-5P. Protist, 162(3), 405-422.

Medlin, L., Elwood, H. J., Stickel, S., & Sogin, M. L. (1988). The characterization of enzymatically amplified eukaryotic 16S-like rRNA-coding regions. *Gene*, *71*(2), 491-499.

**Scholin, C. A**., Villac, M. C., Buck, K. R., Krupp, J. M., Powers, D. A., Fryxell, G. A., & Chavez, F. P. (1994a). Ribosomal DNA sequences discriminate among toxic and non‐toxic *Pseudo-nitzschia* species. Natural toxins, 2(4), 152-165.

**Scholin, C. A**., Herzog, M., Sogin, M., & Anderson, D. M. (1994b). Identification of group‐and strain‐specific genetic markers for globally distributed *Alexandrium* (Dinophyceae). ii. sequence analysis of a fragment of the LSU rRNA gene 1. Journal of phycology, 30(6), 999-1011.

**Stoeck, T.,** Bass, D., Nebel, M., Christen, R., Jones, M. D., Breiner, H. W., & Richards, T. A. (2010). Multiple marker parallel tag environmental DNA sequencing reveals a highly complex eukaryotic community in marine anoxic water. *Molecular ecology*, *19*, 21-31.

**Table S2 -** General characteristic of AGSB-0131 based on the description of species by Puigserver & Zingone, 2002.

| **Cell shape** |  | Oval to Round |
| --- | --- | --- |
| **Cell size** |  |  |
|  | **Length** | 7.12 µm |
|  | **Width** | 6.06 µm |
| **Periflagellar Area** |  |  |
|  | **Shape** |  |
|  | **Length** | 1.91 µm |
|  | **Width** | 1.25 µm |
|  | **Wing-shaped spine** | no |
|  | **No. of platelets** | 1, 2, 3, 4, 5, 6a, 6b, 7(?) & 8 |
|  | **Flagellar Pore** | Yes |
|  | **Accessory Pore** | Yes |
| **Theca ornamentation** |  | Smooth, no ornamentations |
| **Pore pattern** |  |  |
|  | **Large pores** | 4 (up to 6) pores on ventral valve  3 pores on dorsal valve |
|  | **Small pores** | 2 pores on ventral valve (up to 3 on some cells)  2 pores on dorsal valve |
|  | **Marginal pores** | 3 or 4 pores on the ventral valve  4 pores on the dorsal valve |
|  | **Plate centre** |  |
| **Intercalary band sutures** |  | Yes (1.09 µm average sutures) |
| **Pyrenoid** |  | Yes |
| **Trichocysts** |  | Present |

**Table S3** - Assignment of the V4 region (add length) for *P. nux* from using the metaPR2 database. The table shows the ASV retrieved with at least 99% ID.

| **ASV Code** | **% ID** | **reads #** | **Mismatch** | **gaps** | **query start** | **query end** | **ASV start** | **ASV end** | **Division** | **Subdivision** | **Class** | **Order** | **Family** | **Genus** | **Species** |
| --- | --- | --- | --- | --- | --- | --- | --- | --- | --- | --- | --- | --- | --- | --- | --- |
| b3327903cf | 100 | 5583 | 0 | 0 | 21 | 401 | 1 | 381 | Alveolata | Dinoflagellata | Dinophyceae | Prorocentrales | Prorocentraceae | Prorocentrum | Prorocentrum_shikokuense |
| 0ba08de226 | 99.3 | 96160 | 3 | 0 | 8 | 419 | 1 | 412 | Alveolata | Dinoflagellata | Dinophyceae | Dinophyceae_X | Dinophyceae_XX | Dinophyceae_XXX | Dinophyceae_XXX_sp. |
| efdbd39e9f | 99.3 | 37012 | 3 | 0 | 8 | 419 | 1 | 412 | Alveolata | Dinoflagellata | Dinophyceae | Prorocentrales | Prorocentraceae | Prorocentrum | Prorocentrum_mexicanum |
| 045fafe34f | 99.3 | 3749 | 3 | 0 | 1 | 419 | 1 | 419 | Alveolata | Dinoflagellata | Dinophyceae | Peridiniales | Amphidomataceae | Azadinium | Azadinium_trinitatum |
| 3178dd7b6a | 99.3 | 575 | 3 | 0 | 1 | 419 | 2 | 420 | Alveolata | Dinoflagellata | Dinophyceae | Prorocentrales | Prorocentraceae | Prorocentrum | Prorocentrum_shikokuense |

**Table S4** - Assignment of the V9 region (add length) for *P. nux* from using the metaPR2 database. The table shows the ASV retrieved with at least 99% ID.

| **ASV Code** | **% ID** | **reads #** | **Mismatch** | **gaps** | **query start** | **query end** | **ASV start** | **ASV end** | **Division** | **Subdivision** | **Class** | **Order** | **Family** | **Genus** | **Species** |
| --- | --- | --- | --- | --- | --- | --- | --- | --- | --- | --- | --- | --- | --- | --- | --- |
| 602b622101 | 100 | 99575 | 0 | 0 | 33 | 161 | 1 | 129 | Alveolata | Dinoflagellata | Dinophyceae | Peridiniales | Heterocapsaceae | Heterocapsa | Heterocapsa_niei |
| 31c96745e9 | 99.2 | 145270 | 1 | 0 | 33 | 161 | 1 | 129 | Alveolata | Dinoflagellata | Dinophyceae | Gymnodiniales | Kareniaceae | Kareniaceae_X | Kareniaceae_X_sp. |
| 416a29cb7a | 99.2 | 17558 | 0 | 1 | 33 | 161 | 1 | 130 | Alveolata | Dinoflagellata | Dinophyceae | Peridiniales | Heterocapsaceae | Heterocapsa | Heterocapsa_niei |
| 12399335be | 99.2 | 1402 | 1 | 0 | 33 | 161 | 1 | 129 | Alveolata | Dinoflagellata | Dinophyceae | Prorocentrales | Prorocentraceae | Prorocentrum | Prorocentrum_gracile |


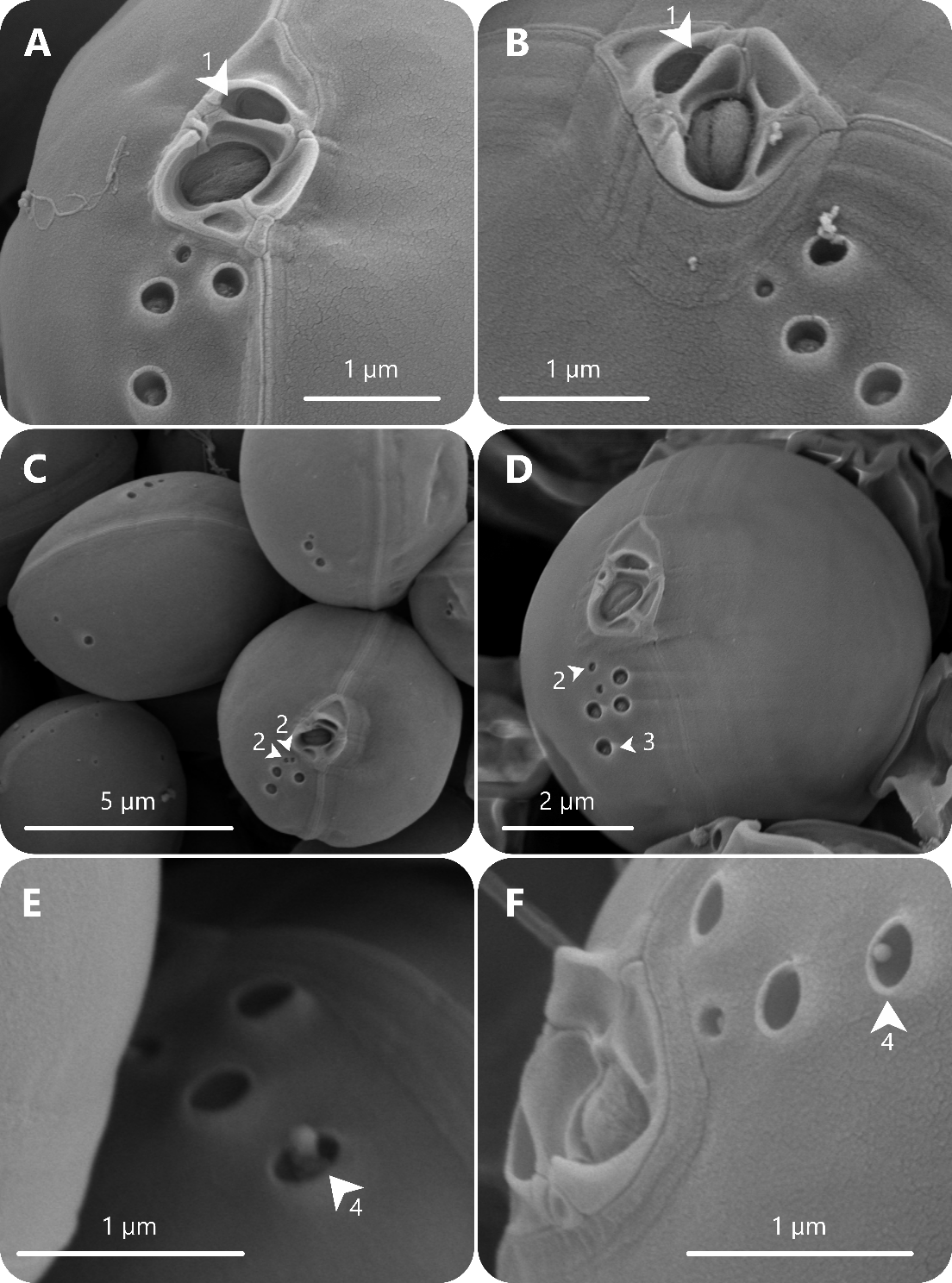
**Figure S1** – SEM images of *P. nux* (AGSB-0131) showing **A** & **B** a view of small platelet 7 (see arrow annotated as n°**1**), partially covered/hidden by platelet 1; **C** & **D** the only two examples of cells observed in SEM with unusual variations in apical pore patterns and numbers (see arrow n°**2** showing an additional small pores and arrow n°**3** pointing at additional large pores) in the right apical portion of the ventral valve. SEM pictures **E** & **F** show trichocysts presence (shown by arrow n°**4**).


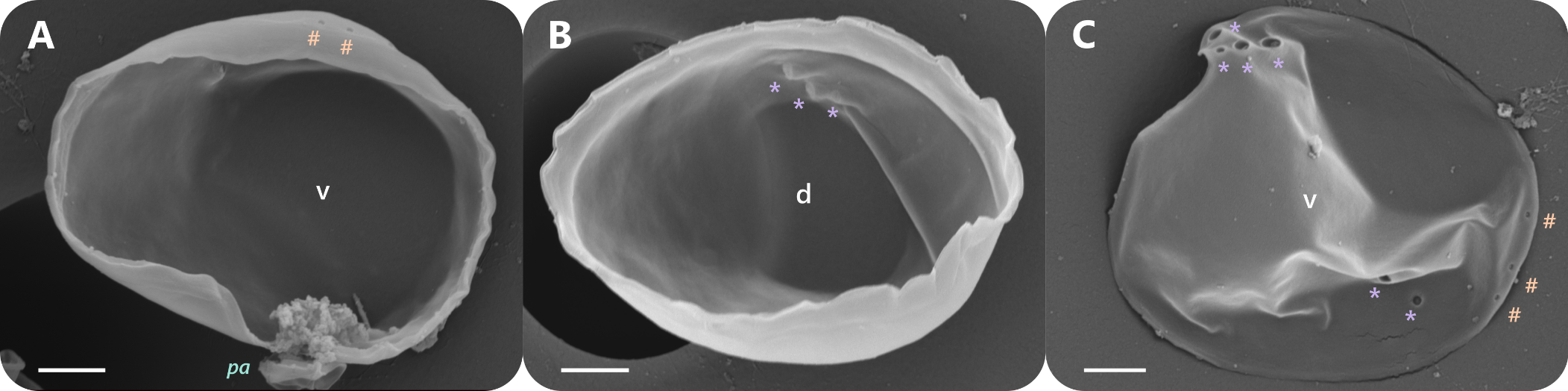


**Figure S2** – SEM images of *P. nux* (AGSB-0131) showing **A** an inside view of an empty ventral (v) valve, **B** an inside view of an empty dorsal (d) valve and **C** external view of a detached ventral valve. Pores were indicated with an asterisk and marginal pores with a sharp sign (#). Periflagellate area was annotated as “pa”. Scale bar is 1 µm.


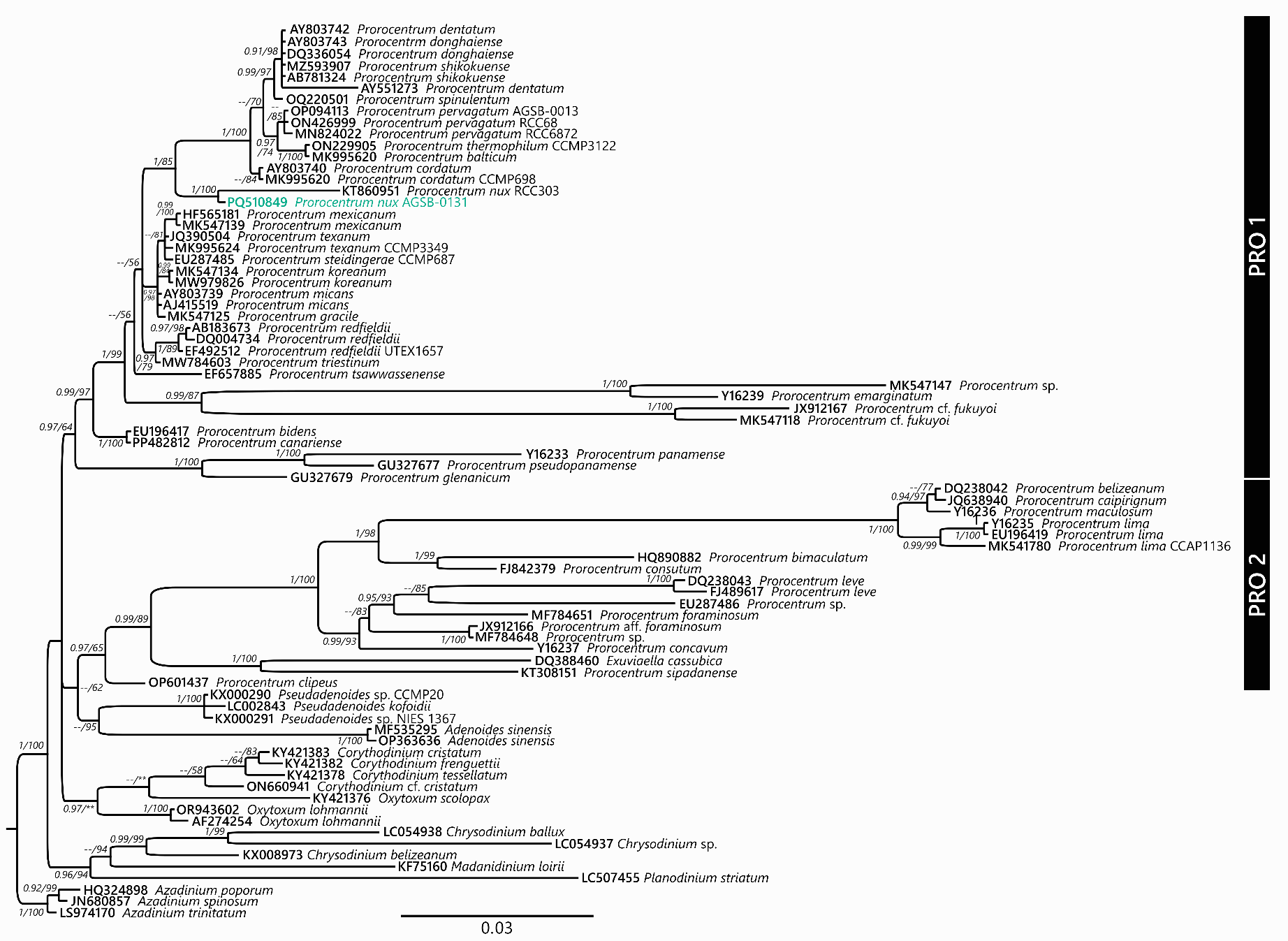


**Figure S3** - Molecular phylogeny of prorocentralean dinophytes including the genus *Prorocentrum* based on the alignment of 18S rRNA. The strain AGSB-0131 sequenced in this study is shown with the associated GenBank Accession Number in green. Corresponding iQtree bootstraps values were added to the tree at each node alongside Bayesian inferences. Bayesian support values and iQtree bootstraps values under 0.90 and 50, respectively, were annotated as -- and a different topology in the tree was annotated with **. For this phylogeny, sequences of *Azadinium poporum* (HQ324898), *Azadinium spinosum* (JN680857) and *Azadinium trinitatum* (LS974170) were used as outgroup.


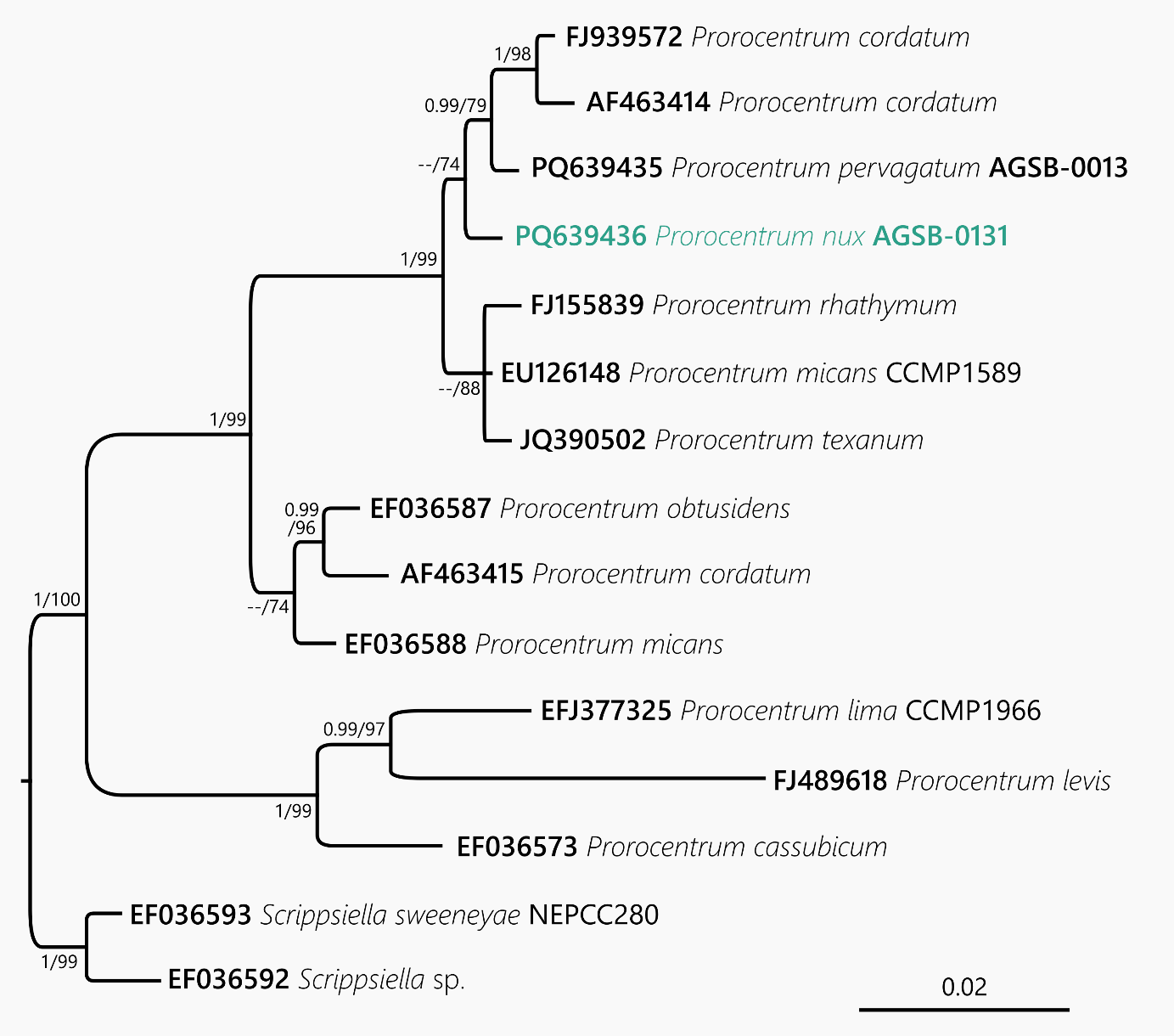


**Figure S4** - Molecular phylogeny of the genus *Prorocentrum* based on the alignment of (COI) mitochondrial gene. The strain AGSB-0131 sequenced in this study is shown with the associated GenBank Accession Number in green. Corresponding RAxML bootstraps were added to the tree at each node alongside Bayesian inferences. Bayesian support values and ML bootstraps under 0.90 and 50, respectively, are annotated as --. For this phylogeny, sequences of *Scrippsiella sweeneyae* (EF036593) and *Scrippsiella* sp. (EF036592) were used as outgroup.


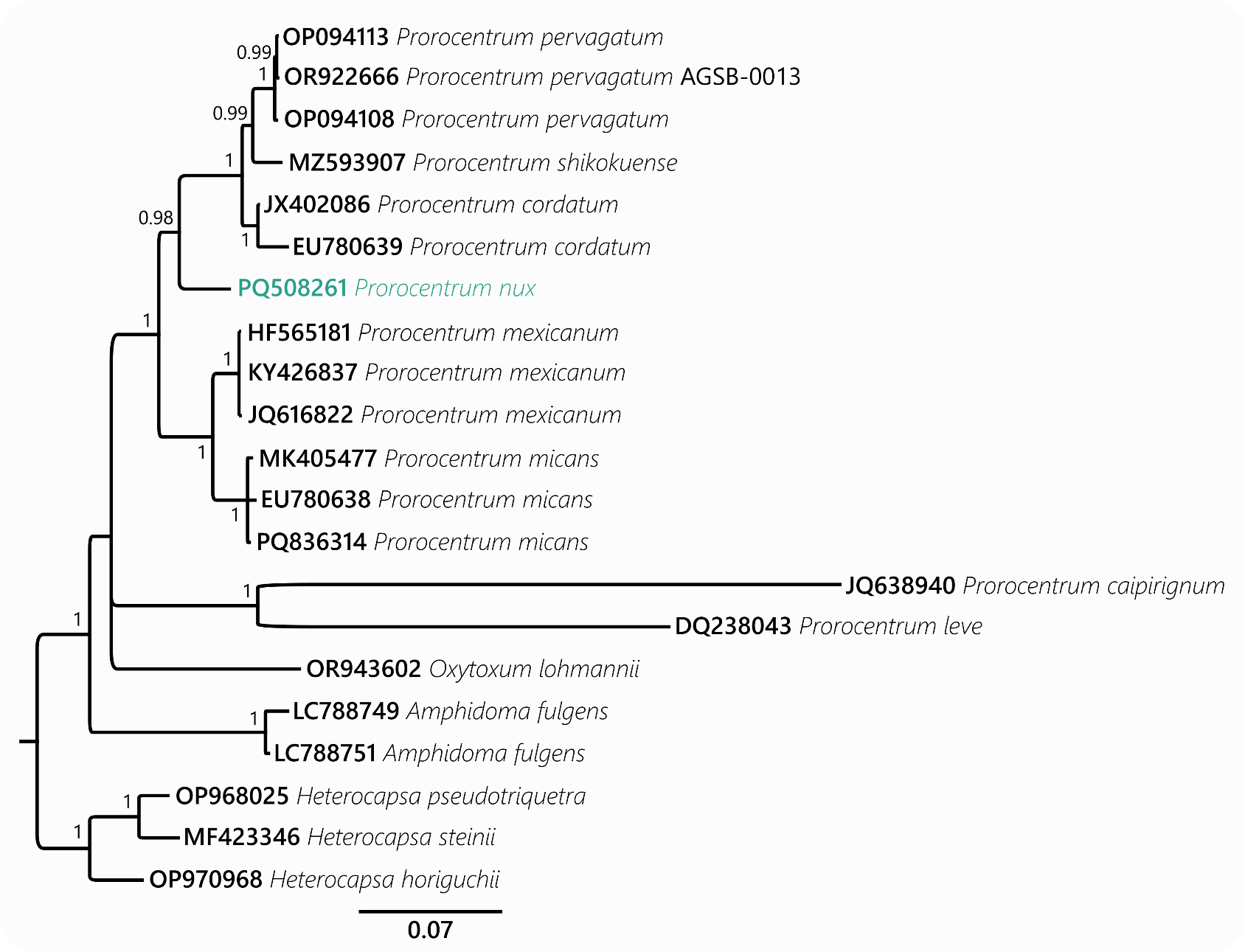


**Figure S5** – Bayesian Concatenated phylogeny of the genus *Prorocentrum* based on the alignment of the 18S, ITS1, 5.8S, ITS2 and 28S rRNA. The strain AGSB-0131 sequenced in this study is shown with the associated GenBank Accession Number in green. Bayesian support values under 0.90 were not shown in the phylogeny. For this phylogeny, sequences of close related genera *Oxytoxum* and *Amphidoma* were also added. *Heterocapsa pseudotriquetra* (OP968025), *Heterocapsa steinii* (MF423346) and *Heterocapsa horiguchii* (OP970968) were used as outgroup.


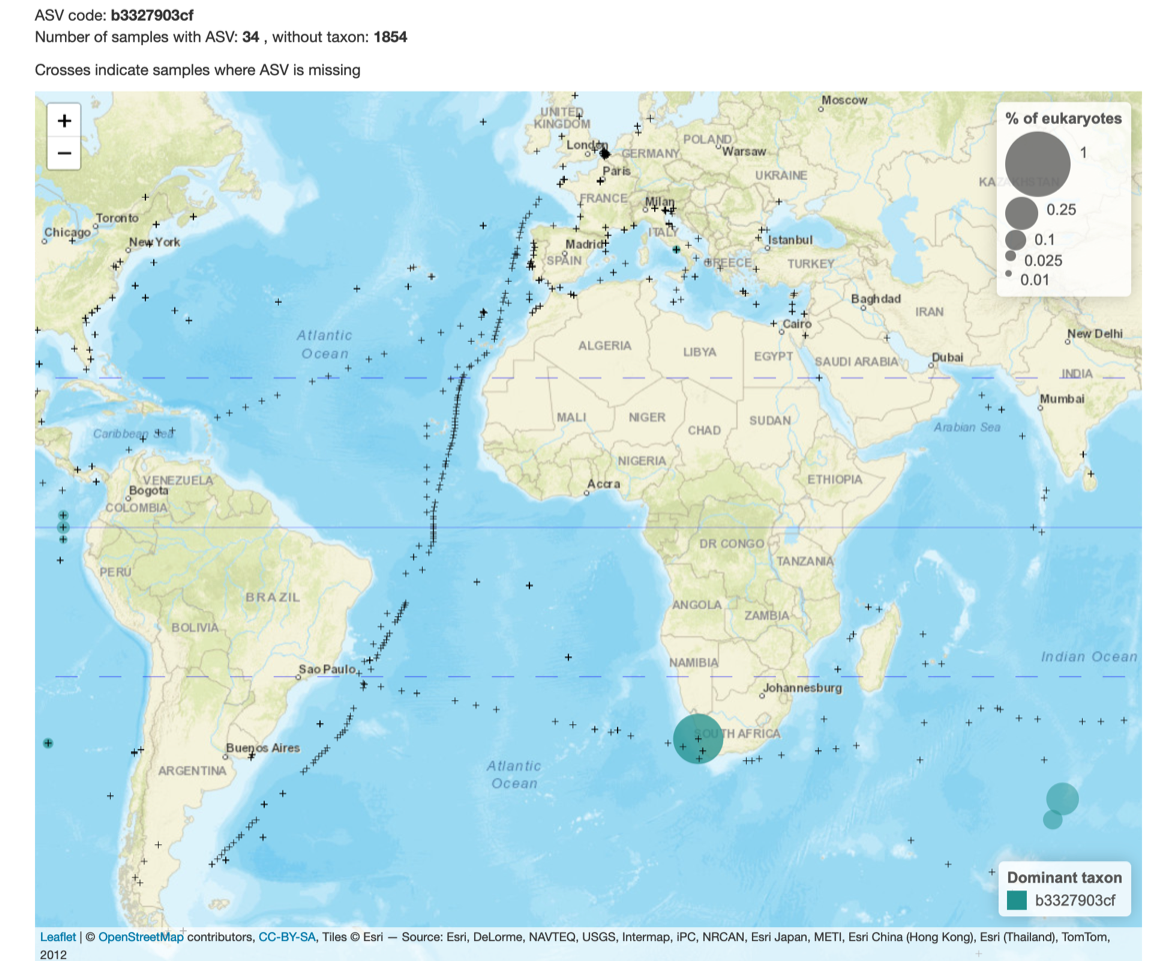


**Figure S6** - Distribution of the amplicon sequence variant ASV_b3327903cf (V4 region) from the metaPR^2^ database (Vaulot et al., 2022). ASV_b3327903cf shown 100% similar with the V4 region from *P. nux*.


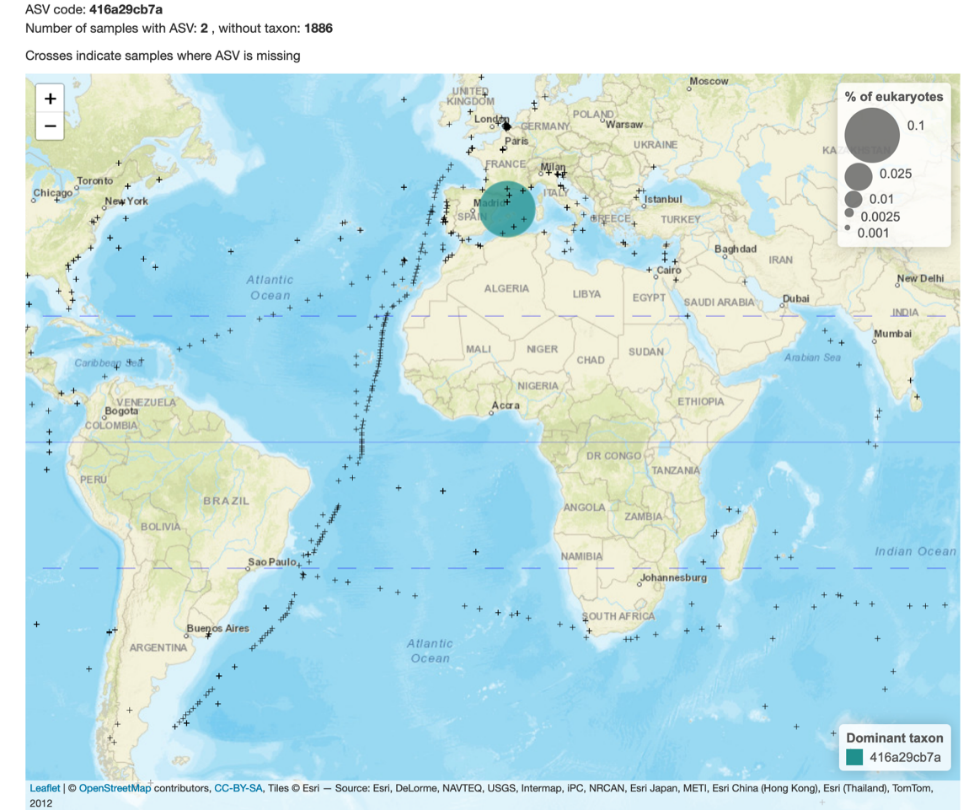

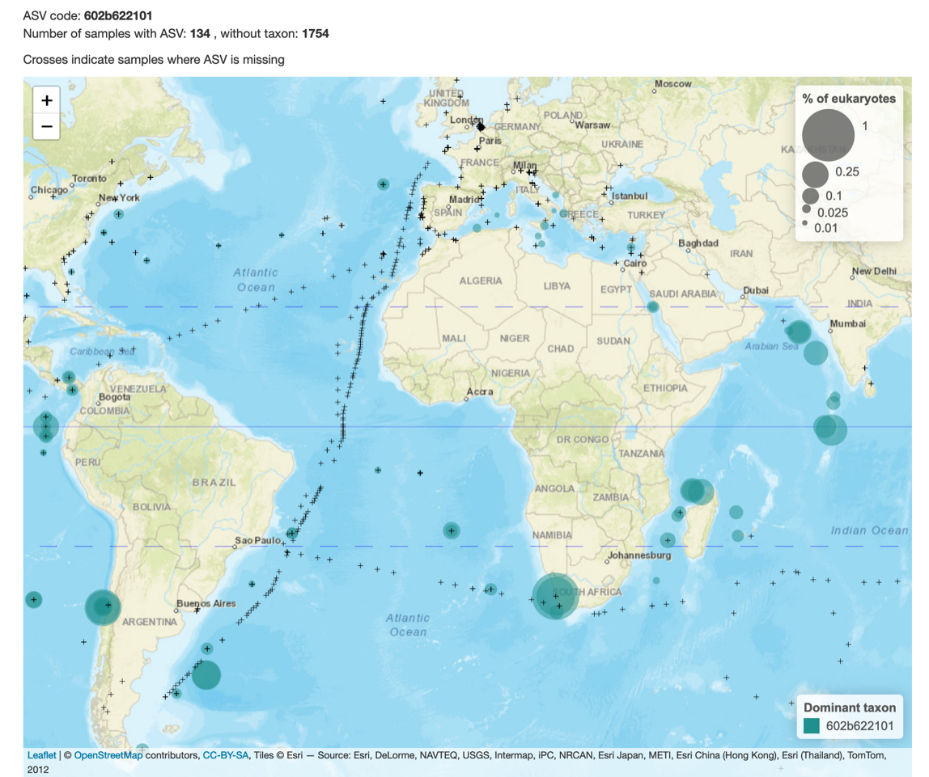


**Figure S7** - Distribution of the amplicon sequence variants a) ASV_602b622101 and b) ASV_416a29cb7a from the metaPR^2^ database (Vaulot et al., 2022). Both ASVs have 0 mismatch with the V9 region from *P. nux*.
